# Interpretability With Accurate Small Models

**DOI:** 10.3389/frai.2020.00003

**Published:** 2020-02-25

**Authors:** Abhishek Ghose, Balaraman Ravindran

**Affiliations:** ^1^Department of Computer Science and Engineering, IIT Madras, Chennai, India; ^2^Department of Computer Science and Engineering, Robert Bosch Centre for Data Science and AI, IIT Madras, Chennai, India

**Keywords:** ML, interpretable machine learning, Bayesian optimization, infinite mixture models, density estimation

## Abstract

Models often need to be constrained to a certain size for them to be considered interpretable. For example, a decision tree of depth 5 is much easier to understand than one of depth 50. Limiting model size, however, often reduces accuracy. We suggest a practical technique that minimizes this trade-off between interpretability and classification accuracy. This enables an arbitrary learning algorithm to produce highly accurate small-sized models. Our technique identifies the training data distribution to learn from that leads to the highest accuracy for a model of a given size. We represent the training distribution as a combination of sampling schemes. Each scheme is defined by a parameterized probability mass function applied to the segmentation produced by a decision tree. An Infinite Mixture Model with Beta components is used to represent a combination of such schemes. The mixture model parameters are learned using Bayesian Optimization. Under simplistic assumptions, we would need to optimize for *O*(*d*) variables for a distribution over a *d*-dimensional input space, which is cumbersome for most real-world data. However, we show that our technique significantly reduces this number to a *fixed set of eight variables* at the cost of relatively cheap preprocessing. The proposed technique is flexible: it is *model-agnostic*, i.e., it may be applied to the learning algorithm for any model family, and it admits a general notion of model size. We demonstrate its effectiveness using multiple real-world datasets to construct decision trees, linear probability models and gradient boosted models with different sizes. We observe significant improvements in the F1-score in most instances, exceeding an improvement of 100% in some cases.

## 1. Introduction

As Machine Learning (ML) becomes pervasive in our daily lives, there is an increased desire to know how models reach specific decisions. In certain contexts this might not be important as long as the ML model itself works well, e.g., in product or movie recommendations. But for certain others, such as medicine and healthcare (Caruana et al., 2015; Ustun and Rudin, 2016), banking^1^, defense applications^2^, and law enforcement^3^ model transparency is an important concern. Very soon, regulations governing digital interactions might necessitate interpretability (Goodman and Flaxman, 2017).

All these factors have generated a lot of interest around “model understanding.” Approaches in the area may be broadly divided into two categories:

1. *Interpretability*: build models that are inherently easy to interpret, e.g., rule lists (Letham et al., 2013; Angelino et al., 2017), decision trees (Breiman et al., 1984; Quinlan, 1993, 2004), sparse linear models (Ustun and Rudin, 2016), decision sets (Lakkaraju et al., 2016), pairwise interaction models that may be linear (Lim and Hastie, 2015), or additive (Lou et al., 2013).
2. *Explainability*: build tools and techniques that allow for explaining black box models, e.g., locally interpretable models such as LIME, Anchors (Ribeiro et al., 2016, 2018), visual explanations for Convolutional Neural Networks such as Grad-CAM (Selvaraju et al., 2017), influence functions (Koh and Liang, 2017), feature attribution based on Shapley values (Lundberg and Lee, 2017; Ancona et al., 2019).

Our work addresses the problem of interpretability by providing a way to increase accuracy of existing models that are considered interpretable.

Interpretable models are preferably small in *size*: this is referred to as low *explanation complexity* in Herman (2017), is seen as a form of *simulability* in Lipton (2018), is a motivation for *shrinkage methods* (Hastie et al., 2009, section 3.4), and is often otherwise listed as a desirable property for interpretable models (Lakkaraju et al., 2016; Ribeiro et al., 2016; Angelino et al., 2017). For instance, a decision tree of *depth* = 5 is easier to understand than one of *depth* = 50. Similarly, a linear model with 10 non-zero terms might be easier to comprehend than one with 50 non-zero terms. This indicates an obvious problem: an interpretable model is often small in its size, and since model size is usually inversely proportional to the bias, a model often sacrifices accuracy for interpretability.

We propose a technique to minimize this tradeoff for any model family; thus our approach is *model agnostic*. Our technique adaptively samples the provided training data, and identifies a sample on which to learn a model of a given size; the property of this sample being that it is optimal in terms of the accuracy of the constructed model. What makes this strategy practically valuable is that the accuracy of this model may often be significantly higher than one learned on the training data as-is, especially when the model size is small.

Let,

1. *accuracy*(*M, p*) be the classification accuracy of model *M* on data represented by the joint distribution *p*(*X, Y*) of instances *X* and labels *Y*. We use the term “accuracy” as a generic placeholder for a measure of model correctness. This may specifically measure *F1-score, AUC, lift*, etc., as needed.
2. $train_{F}\left(p,\eta \right)$ produce a model obtained using a specific training algorithm, e.g., CART (Breiman et al., 1984), for a given model family F, e.g., decision trees, where the model size is fixed at η, e.g., trees with *depth* = 5. The training data is represented by the joint distribution *p*(*X, Y*) of instances *X* and labels *Y*.

If we are interested in learning a classifier of size η for data with distribution *p*(*X, Y*), our technique produces the *optimal training distribution* $p_{\eta }^{*}\left(X,Y\right)$ such that:

(1) $$\begin{array}{r} p_{\eta }^{*}=\underset{q}{\arg\;\max}accuracy\left(train_{F}\left(q,\eta \right),p\right) \end{array}$$

Here *q*(*X, Y*) ranges over all possible distributions over the data (*X, Y*).

Training a model on this optimal distribution produces a model that is at least as good as training on the original distribution *p*:

(2) $$\begin{array}{r} accuracy\left(train_{F}\left(p,\eta \right),p\right)\leq accuracy\left(train_{F}\left(p_{\eta }^{*},\eta \right),p\right) \end{array}$$

Furthermore, the relationship in Equation (2) may be separated into two regimes of operation. A model trained on $p_{\eta }^{*}$ outperforms one trained on the original distribution *p* up to a model size η′, with both models being comparably accurate beyond this point:

(3) $$\begin{array}{r} \text{For}\;\eta \leq \eta^{\prime },accuracy\left(train_{F}\left(p,\eta \right),p\right)<accuracy\left(train_{F}\left(p_{\eta }^{*},\eta \right),p\right) \end{array}$$

(4) $$\begin{array}{r} \text{For}\;\eta >\eta^{\prime },accuracy\left(train_{F}\left(p,\eta \right),p\right)=accuracy\left(train_{F}\left(p_{\eta }^{*},\eta \right),p\right) \end{array}$$

Our key contributions in this work are:

1. Postulating that the optimal training distribution may be different than the test distribution. This challenges the conventional wisdom that the training and test data must come from the same distribution, as in the LHS of Equations (2), (3), and (4).
2. Providing a model-agnostic and practical adaptive sampling based technique that exploits this effect to learn small models, that often possess higher accuracy compared to using the original distribution.
3. Demonstrating the effectiveness of our technique with different learning algorithms, $train_{F}\left(\;\right)$, and multiple real world datasets. Note that our benchmark is not a specific algorithm that learns small models; the value of our approach is in its being *model-agnostic*: it works with arbitrary learners.
4. We show that learning the distribution, $p_{\eta }^{*}$, in the *d* dimensions of the data, may be decomposed into a relatively cheap preprocessing step that depends on *d*, followed by a core optimization step *independent* of *d*: the optimization is over a fixed set of eight variables. This makes our technique scalable.

We do not impose any constraints on the specification of $train_{F}\left(\;\right)$ for it to create interpretable models; our technique may be used with any model family. But the fact that we see increased accuracy *up to* a model size (η′ in Equation 3), makes the technique *useful* in setups where small sized models are preferred. Applications requiring interpretability are an example of this. There may be others, such as *model compression*, which we have not explored, but briefly mention in section 5.2.

## 2. Overview

This section provides an overview of various aspects of our work: we impart some intuition for why we expect the train and test distributions to differ for small-sized models, describe where our technique fits into a model building workflow, mention connections to previous work and establish our notation and terminology.

### 2.1. Intuition

**Figure 1:**
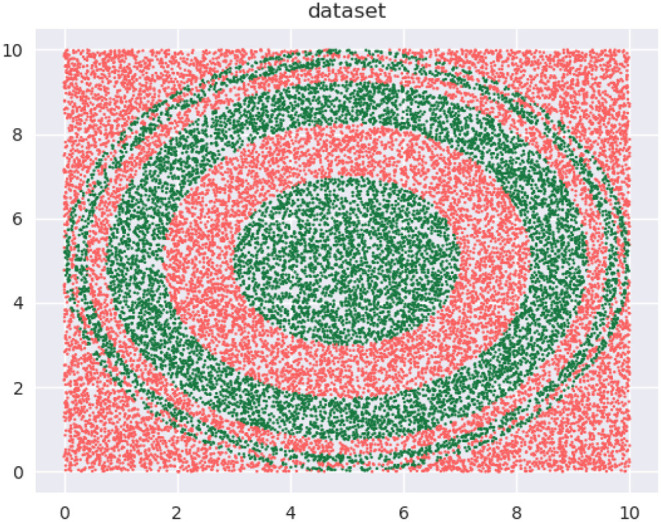
Binary class dataset for classification.

Let's begin with a quick demonstration of how modifying the training distribution can be useful. We have the binary class data, shown in Figure 1, that we wish to classify with decision trees with *depth* = 5.

**Figure 2:**
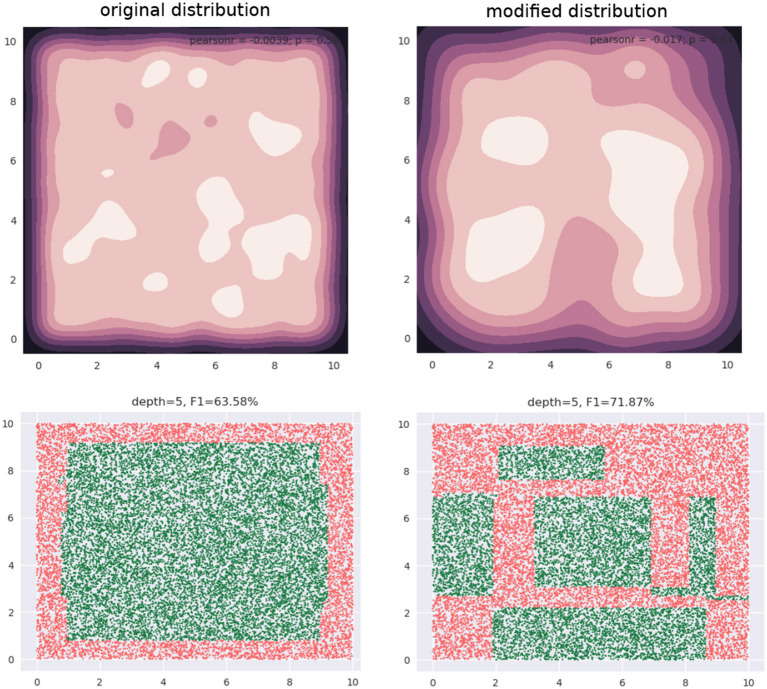
Changing the input distribution can significantly affect model accuracy.

Our training data is a subset of this data (not shown). The training data is approximately uniformly distributed in the input space—see the 2D *kernel density* plot in the top-left panel in Figure 2. The bottom-left panel in the figure shows the regions of a decision tree with *depth* = 5 learns, using the CART algorithm. The top-right panel shows a modified distribution of the data (now the density seems to be relatively concentrated away from the edge regions of the input space), and the corresponding decision tree with *depth* = 5, also learned using CART, is visualized in the bottom-right panel. Both decision trees used the same learning algorithm and possess the same depth. As we can see, the *F*1 scores are significantly different: 63.58 and 71.87%, respectively.

Where does this additional accuracy come from?

All classification algorithms use some heuristic to make learning tractable, e.g.,:

- Decision Trees—one step lookahead (note that the CART tree has a significantly smaller number of leaves than the possible 2^5^ = 32, in our example).
- Logistic Regression—local search, e.g., *Stochastic Gradient Descent (SGD)*.
- Artificial Neural Networks (ANN)—local search, e.g., SGD, *Adam*.

Increasing the size allows for offsetting the shortcomings of the heuristic by adding parameters to the model till it is satisfactorily accurate: increasing depth, terms, hidden layers, or nodes per layer. Our hypothesis is, in restricting a model to a small size, this potential gap between the *representational* and *effective* capacities becomes pronounced. In such cases, modifying the data distribution guides the heuristic to focus learning on regions of the input space that are valuable in terms of accuracy. We are able to empirically demonstrate this effect for DTs in section 4.2.1.

### 2.2. Workflow

**Figure 3:**
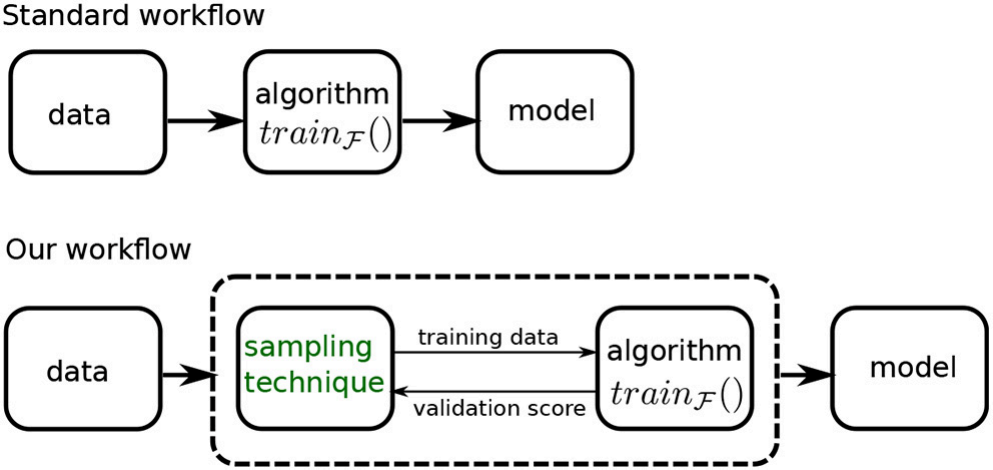
Our workflow compared with the standard workflow.

Figure 3 shows how our sampling technique modifies the model building workflow. In the standard workflow, we feed the data into a learning algorithm, $train_{F}\left(\;\right)$, to obtain a model. In our setup, the data is presented to a system, represented by the dashed box, that is comprised of both the learning algorithm and our sampling technique.

This system produces the final model in an iterative fashion: the sampling technique (or *sampler*) produces a sample using its current distribution parameters, that is used by the learning algorithm to produce a model. This model is evaluated on a validation dataset and the validation score is conveyed back to the sampler. This information is used to modify the distribution parameters and generate a new training sample for the algorithm, and so on, till we reach a stopping criteria. The criteria we use is a specified number of iterations—we refer to this as our *budget*. The best model produced within the budget, as measured by the validation score, is our final model, and the corresponding distribution is presented as the ideal training distribution.

### 2.3. Previous Work

We are aware of no prior work that studies the relationship between data distribution and accuracy in the small model regime. In terms of the larger view of modifying the training distribution to influence learning, parallels may be drawn to the following methodologies:

1. When learning on data with class imbalance, using a different train distribution compared to test via over/under-sampling (Japkowicz and Stephen, 2002), is a commonly used strategy. Seen from this perspective, we are positing that modifying the original distribution is helpful in a wider set of circumstances, i.e., when there is no imbalance, as in Figure 1, but the model is restricted in size.
2. Among popular techniques, *Active Learning* (Settles, 2009; Dasgupta, 2011) probably bears the strongest resemblance to our approach. However, our problem is different in the following key respects:
  - In active learning, we don't know the labels of most or all of the data instances, and there is an explicit label acquisition cost that must be accounted for. In contrast, our work targets the traditional supervised learning setting where the joint distribution of instances and labels is approximately known through a fixed set of samples drawn from that distribution.
  - Because there is a label acquisition cost, learning from a small subset of the data such that the resulting model approximates one learned on the complete dataset, is strongly incentivized. This economy in sample size is possibly the most common metric used to evaluate the utility of an active learner. This is different from our objective, where we are not interested in minimizing training data size, but in learning small-sized models. Further, we are interested in *outperforming* a model learned on the complete data.
3. *Coreset* construction techniques (Bachem et al., 2017; Munteanu and Schwiegelshohn, 2018) seek to create a “summary” weighted sample of a dataset with the property that a model learned on this dataset approximates one learned on the complete dataset. Here too, the difference in objectives is that we focus on small models, ignore training data size, and are interested in outperforming a model learned on the complete data.

This is not to say that the tools of analysis from the areas of active learning or coreset identification cannot be adapted here; but current techniques in these areas do not solve for our objective.

### 2.4. Terminology and Notation

Let's begin with the notion of “model size.” Even though there is no standard notion of size across model families, or even within a model family, we assume the term informally denotes model attribute(s) with the following properties:

1. *size* ∝ *bias*^−1^
2. Smaller the size of a model, easier it is to interpret.

As mentioned earlier, only property 1 is strictly required for our technique to be applicable; property 2 is needed for interpretability.

Some examples of model size are depth of decision trees, number of non-zero terms in a linear model and number of rules in a rule set.

In practice, a model family may have *multiple* notions of size depending upon the modeler, e.g., depth of a tree or the number of leaves. The size might even be determined by multiple attributes in conjunction, e.g., maximum depth of each tree *and* number of boosting rounds in the case of a *gradient boosted model (GBM)*. It is also possible that while users of a model might agree on a definition of size they might disagree on the *value* for the size up to which the model stays interpretable. For example, are decision trees interpretable up to a depth of 5 or 10? Clearly, the definition of size and its admissible values might be subjective. Regardless, the discussion in this paper remains valid as long as the notion of size exhibits the properties above. With this general notion in mind, we say that interpretable models are typically *small*.

Here are the notations we use:

1. The matrix *X* ∈ ℝ^*N*×*d*^ represents an ordered collection of *N* input feature vectors, each of which has *d* dimensions. We assume individual feature vectors $x_{i}\in {\mathbb{R}}^{d\times 1}$ to be column vectors, and hence the *ith* row of *X* represents $x_{i}^{T}$. We occasionally treat *X* as a set and write *x*_*i*_ ∈ *X* to denote the feature vector *x*_*i*_ is part of the collection *X*.
  An ordered collection of *N* labels is represented by the vector *Y* ∈ ℝ^*N*^.
  We represent a dataset with *N* instances with the tuple (*X, Y*), where *X* ∈ ℝ^*N*×*d*^, *Y* ∈ ℝ^*N*^, and the label for *x*_*i*_ is *Y*_*i*_, where 1 ≤ *i* ≤ *N*.
2. The element at the *p*th row and *q*th column indices of a matrix *A* is denoted by [*A*]_*pq*_.
3. We refer to the joint distribution *p*(*X, Y*) from which a given dataset was sampled, as the *original distribution*. In the context of learning a model and predicting on a held-out dataset, we distinguish between the *train, validation*, and *test* distributions. In this work, the train distribution may or may not be identical to the original distribution, which would be made clear by the context, but the validation and test distributions are *always* identical to the original distribution.
4. The terms *pdf* and *pmf* denote *probability density function* and *probability mass function*, respectively. The term “probability distribution” may refer to either, and is made clear by the context. A distribution *p*, parameterized by θ, defined over the variable *x*, is denoted by *p*(*x*; θ).
5. We use the following terms introduced before:
  - *accuracy*(*M, p*) is the classification accuracy of model *M* on data represented by the joint distribution *p*(*X, Y*) of instances *X* and labels *Y*. We often overload this term to use a dataset instead of distribution. In this case, we write *accuracy*(*M*, (*X, Y*)) where (*X, Y*) is the dataset.
  - $train_{F}\left(p,\eta \right)$ produces a model obtained using a specific training algorithm for a model family F, where the model size is fixed at η. This may also be overloaded to use a dataset, and we write: $train_{F}\left(\left(X,Y\right),\eta \right)$.
6. We denote the depth of a tree *T* by the function *depth*(*T*).
7. ℝ, ℤ, and ℕ denote the sets of *reals, integers*, and *natural numbers*, respectively.

     □

The rest of the paper is organized as follows: in section 3 we describe in detail two formulations of the problem of learning the optimal density. Section 4 reports experiments we have conducted to evaluate our technique. It also presents our analysis of the results. We conclude with section 5 where we discuss some of the algorithm design choices and possible extensions of our technique.

## 3. Methodology

In this section we describe our sampling technique. We begin with a intuitive formulation of the problem in section 3.1 to illustrate challenges with a simple approach. This also allows us to introduce the relevant mathematical tools. Based on our understanding here, we propose a much more efficient approach in section 3.3.

### 3.1. A Naive Formulation

We phrase the problem of finding the ideal density (for the learning algorithm) as an optimization problem. We represent the density over the input space with the *pdf* *p*(*x*; Ψ), where Ψ is a parameter vector. Our optimization algorithm runs for a budget of *T* time steps. Algorithm 1 lists the execution steps.

**Algorithm 1:**
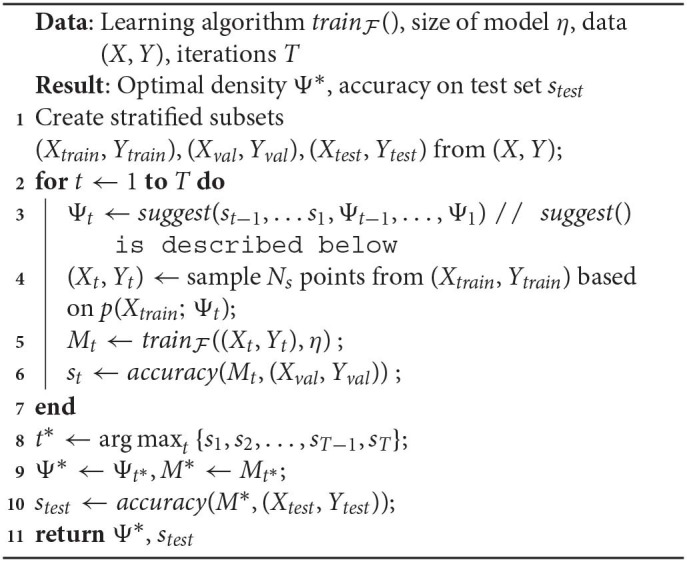
Naive formulation

In Algorithm 1:

1. *suggest*() is a call to the optimizer at time *t*, that accepts past validation scores *s*_*t*−1_, …*s*_1_ and values of the density parameter Ψ_*t*−1_, …, Ψ_1_. These values are randomly initialized for *t* = 1. Note that not all optimizers require this information, but we refer to a generic form of optimization that makes use of the entire history.
2. In Line 4, a sampled dataset (*X*_*t*_, *Y*_*t*_) comprises of instances *x*_*i*_ ∈ *X*_*train*_, and their corresponding labels *y*_*i*_ ∈ *Y*_*train*_. Denoting the sampling weight of an instance *x*_*i*_ as *w*(*x*_*i*_), we use *w*(*x*_*i*_) ∝ *p*(*x*_*i*_; Ψ_*t*_), ∀*x*_*i*_ ∈ *X*_*train*_.
  The sampling in Line 10 is analogous.
3. Although the training happens on a sample drawn based on Ψ_*t*_, the validation dataset (*X*_*val*_, *Y*_*val*_) isn't modified by the algorithm and always reflects the original distribution. Hence, *s*_*t*_ represents the accuracy of a model on the original distribution.
4. In the interest of keeping the algorithm simple to focus on the salient steps/challenges, we defer a discussion of the sample size *N*_*s*_ to our improved formulation in section 3.3.

Algorithm 1 represents a general framework to discover the optimal density within a time budget *T*. We refer to this as a “naive” algorithm, since within our larger philosophy of discovering the optimal distribution, this is the most direct way to do so. It uses *accuracy*() as both the objective and fitness function, where the score *s*_*t*_ is the fitness value for current parameters Ψ_*t*_. It is easy to see here what makes our technique model-agnostic: the arbitrary learner $train_{F}\left(\;\right)$ helps define the fitness function but there are no assumptions made about its form. While conceptually simple, clearly the following key implementation aspects dictate its usefulness in practice:

1. The optimizer to use for *suggest*().
2. The precise representation of the *pdf* *p*(*x*; Ψ).

We look at these next.

#### 3.1.1. Optimization

The fact that our objective function is not only a black-box, but is also noisy, makes our optimization problem hard to solve, especially within a budget *T*. The quality of the optimizer *suggest*() critically influences the utility of Algorithm 1.

We list below the characteristics we need our optimizer to possess:

1. **Requirement 1: It should be able to work with a black-box objective function**. Our objective function is *accuracy*(), which depends on a model produced by $train_{F}\left(\;\right)$. The latter is an input to the algorithm and we make no assumptions about its form. The cost of this generality is that *accuracy*() is a black-box function and our optimizer needs to work without knowing its smoothness, amenability to gradient estimation etc.
2. **Requirement 2: Should be robust against noise**. Results of *accuracy*() may be noisy. There are multiple possible sources of noise, e.g.,:
  1. The model itself is learned on a sample (*X*_*t*_, *y*_*t*_).
  2. The classifier might use a local search method like SGD whose final value for a given training dataset depends on various factors like initialization, order of points, etc.
3. **Requirement 3: Minimizes calls to the objective function**. The acquisition cost of a fitness value *s*_*t*_ for a solution Ψ_*t*_ is high: this requires a call to *accuracy*(), which in turn calls $train_{F}\left(\;\right)$. Hence, we want the optimizer to minimize such calls, instead shifting the burden of computation to the optimization strategy. The number of allowed calls to *accuracy*() is often referred to as the *fitness evaluation budget*.

Some optimization algorithms that satisfy the above properties to varying degrees are the class of *Bayesian Optimization (BO)* (Brochu et al., 2010; Shahriari et al., 2016) algorithms; evolutionary algorithms such as *Covariance Matrix Adaptation Evolution Strategy (CMA-ES)* (Hansen and Ostermeier, 2001; Hansen and Kern, 2004) and *Particle Swarm Optimization (PSO)* (Kennedy and Eberhart, 1995; Parsopoulos and Vrahatis, 2001); heuristics based algorithms such as *Simulated Annealing* (Kirkpatrick et al., 1983; Gelfand and Mitter, 1989; Gutjahr and Pflug, 1996); bandit-based algorithms such as *Parallel Optimistic Optimization* (Grill et al., 2015) and *Hyperband* (Li L. et al., 2017).

We use BO here since it has enjoyed substantial success in the area of *hyperparameter optimization* (e.g., Bergstra et al., 2011; Snoek et al., 2012; Perrone et al., 2018; Dai et al., 2019), where the challenges are similar to ours.

While a detailed discussion of BO techniques is beyond the scope of this paper (refer to Brochu et al., 2010; Shahriari et al., 2016 for an overview), we briefly describe why they meet our requirements: BO techniques build their own model of the response surface over multiple evaluations of the objective function; this model serves as a *surrogate* (whose form is known) for the actual black-box objective function. The BO algorithm relies on the surrogate alone for optimization, bypassing the challenges in directly working with a black-box function (Requirement 1 above). The surrogate representation is also probabilistic; this helps in quantifying uncertainties in evaluations, possibly arising due to noise, making for robust optimization (Requirement 2). Since every call to *suggest*() is informed by this model, the BO algorithm methodically focuses on only the most promising regions in the search space, making prudent use of its fitness evaluation budget (Requirement 3).

The family of BO algorithms is fairly large and continues to grow (Bergstra et al., 2011; Hutter et al., 2011; Snoek et al., 2012, 2015; Wang et al., 2013; Gelbart et al., 2014; Hernández-Lobato et al., 2016; Levesque et al., 2017; Li C. et al., 2017; Rana et al., 2017; Malkomes and Garnett, 2018; Perrone et al., 2018; Alvi et al., 2019; Dai et al., 2019; Letham et al., 2019; Nayebi et al., 2019). We use the *Tree Structured Parzen Estimator (TPE)* algorithm (Bergstra et al., 2011) since it scales linearly with the number of evaluations (the runtime complexity of a naive BO algorithm is *cubic* in the number of evaluations; see Shahriari et al., 2016) and has a popular and mature library: *Hyperopt* (Bergstra et al., 2013).

#### 3.1.2. Density Representation

The representation of the *pdf*, *p*(*x*; Ψ) is the other key ingredient in Algorithm 1. The characteristics we are interested in are:

1. **Requirement 1: It must be able to represent an arbitrary density function**. This is an obvious requirement since we want to *discover* the optimal density.
2. **Requirement 2: It must have a fixed set of parameters**. This is for convenience of optimization, since most optimizers cannot handle the *conditional parameter spaces* that some *pdf* representations use. A common example of the latter is the popular *Gaussian Mixture Model (GMM)*, where the number of parameters increases linearly with the number of mixture components.
  This algorithm design choice allows for a larger scope of being able to use different optimizers in Algorithm 1; there are many more optimizers that can handle fixed compared to conditional parameter spaces. And an optimizer that works with the latter, can work with a fixed parameter space as well^4^.

The *Infinite Gaussian Mixture Model (IGMM)* (Rasmussen, 1999), a *non-parametric Bayesian* extension to the standard GMM, satisfies these criteria. It side-steps the problem of explicitly denoting the number of components by representing it using a *Dirichlet Process (DP)*. The DP is characterized by a *concentration parameter* α, which determines both the number of components (also known as *partitions* or *clusters*) and association of a data point to a specific component. The parameters for these components are not directly learned, but are instead drawn from prior distributions; the parameters of these prior distributions comprises our fixed set of variables (Requirement 2). We make the parameter α part of our optimization search space, so that the appropriate number of components maybe discovered; this makes our *pdf* flexible (Requirement 1).

We make a few modifications to the IGMM for it to better fit our problem. This doesn't change its compatibility to our requirements. Our modifications are:

1. Since our data is limited to a “bounding box” within ℝ^*d*^ (this region is easily found by determining the *min* and *max* values across instances in the provided dataset, for each dimension, ignoring outliers if needed), we replace the Gaussian mixture components with a multivariate generalization of the *Beta* distribution. We pick *Beta* since it naturally supports bounded intervals. In fact, we may treat the data as lying within the unit hypercube [0, 1]^*d*^ without loss of generality, and with the understanding that the features of an instance are suitably scaled in the actual implementation.
  Using a bounded interval distribution provides the additional benefit that we don't need to worry about infeasible solution regions in our optimization.
2. Further, we assume *independence* across the *d* dimensions as a starting point. We do this to minimize the number of parameters, similar to using a diagonal covariance matrix in GMMs.
  Thus, our *d*-dimensional generalization of the *Beta* is essentially a set of *d* *Beta* distributions, and every component in the mixture is associated with such a set. For *k* mixture components, we have *k*×*d* *Beta* distributions in all, as against *k* *d*-dimensional Gaussians in an IGMM.
3. A *Beta* distribution uses two positive valued *shape parameters*. Recall that we don't want to learn these parameters for each of the *k*×*d* *Beta* distributions (which would defeat our objective of a fixed parameter space); instead we sample these from prior distributions. We use *Beta* distributions for our priors too: each shape parameter is drawn from a corresponding prior *Beta* distribution.
  Since we have assumed that the dimensions are independent, we have two prior *Beta* for the shape parameters *per dimension*. We obtain the parameters {*A*_*j*_, *B*_*j*_} of a *Beta* for dimension *j*, 1 ≤ *j* ≤ *d*, by drawing *A*_*j*_~*Beta*(*a*_*j*_, *b*_*j*_) and $B_{j}\sim Beta\left(a_{j}^{\prime },b_{j}^{\prime }\right)$, where {*a*_*j*_, *b*_*j*_} and $\left\{a_{j}^{\prime },b_{j}^{\prime }\right\}$ are the shape parameters of the priors.
  There are a total of 4*d* prior parameters, with 4 prior parameters $\left\{a_{j},b_{j},a_{j}^{\prime },b_{j}^{\prime }\right\}$ per dimension *j*, 1 ≤ *j* ≤ *d*.

We refer to this mixture model as an *Infinite Beta Mixture Model (IBMM)*^5^. For *d* dimensional data, we have $\text{Ψ}=\left\{\alpha ,a_{1},b_{1},a_{1}^{\prime },b_{1}^{\prime },\ldots ,a_{d},b_{d},a_{d}^{\prime },b_{d}^{\prime }\right\}$. This is a total of 4*d* + 1 parameters.

Algorithm 2 shows how we sample *N*_*t*_ points from (*X, Y*) using the IBMM.

**Algorithm 2:**
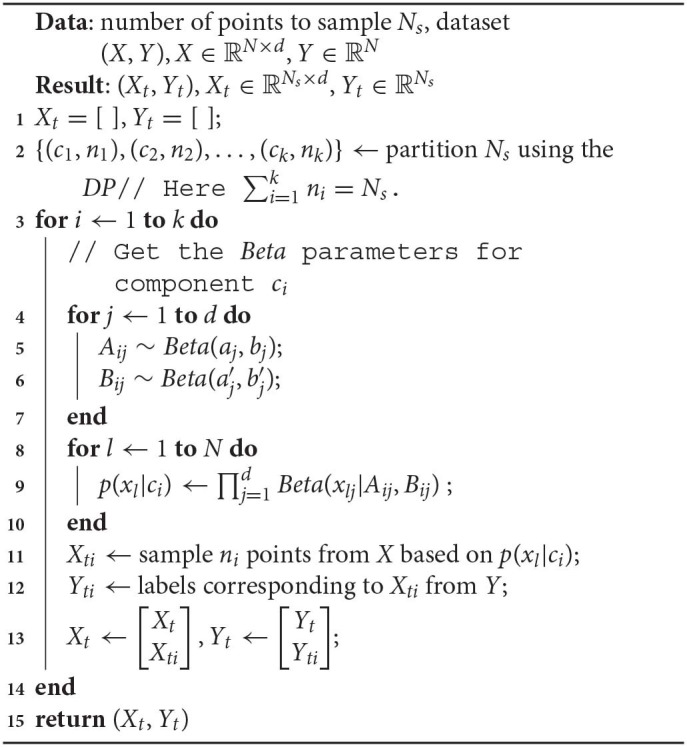
Sampling using IBMM

We first determine the partitioning of the number *N*_*s*_, induced by the *DP* (line 2). We use *Blackwell-MacQueen* sampling (Blackwell and MacQueen, 1973) for this step. This gives us *k* components, denoted by *c*_*i*_, 1 ≤ *i* ≤ *k*, and the corresponding number of points *n*_*i*_, 1 ≤ *i* ≤ *k* to be assigned to each component. We then sample points one component at a time: we draw the *Beta* parameters per dimension—*A*_*ij*_, *B*_*ij*_—from the priors (lines 4–6), followed by constructing sampling weights *p*(*x*_*l*_|*c*_*i*_), ∀*x*_*l*_ ∈ *X* assuming independent dimensions (line 9).

We emphasize here that we use the IBMM *purely for representational convenience*. All the 4*d* + 1 parameters are learned by the optimizer, and we ignore the standard associated machinery for estimation or inference. These parameters *cannot* be learned from the data since our fundamental hypothesis is that the optimal distribution is different from the original distribution.

### 3.2. Challenges

The primary challenge with this formulation is the size of the search space. We have successfully tried out Algorithm 1 on small toy datasets as proof-of-concept, but for most real world datasets, optimizing over 4*d* + 1 variables leads to an impractically high run-time even using a fast optimizer like TPE.

One could also question the independence assumption for dimensions, but that doesn't address the problem of the number of variables: learning a *pdf* directly in *d* dimensions would require *at least* *O*(*d*) optimization variables. In fact, a richer assumption makes the problem worse with *O*(*d*^2^) variables to represent inter-dimension interactions.

### 3.3. An Efficient Approach Using Decision Trees

We begin by asking if we can prune the search space in some fashion. Note that we are solving a *classification* problem, measured by *accuracy*(); however, the IBMM only indirectly achieves this goal by searching the complete space Ψ. The search presumably goes through distributions with points from only one class, no points close to any or most of the class boundary regions, etc; distributions that decidedly result in poor fitness scores. Is there a way to exclude such “bad” configuration values from the search space?

One strategy would be to first determine where the class boundaries lie, and *penalize* any density Ψ_*t*_ that doesn't have at least some overlap with them. This is a common optimization strategy used to steer the search trajectory away from bad solutions. However, implementation-wise, this leads to a new set of challenges:

1. How do we determine, and then represent, the location of class boundaries?
2. What metric do we use to appropriately capture our notion of overlap of Ψ_*t*_ and these locations?
3. How do we efficiently execute the previous steps? After all, our goal is to either (a) reduce the number of optimization variables OR (b) significantly reduce the size of the search space for the current *O*(*d*) variables.

We offer a novel resolution to these challenges that leads to an efficient algorithm by making the optimization “class boundary sensitive.”

**Figure 4:**
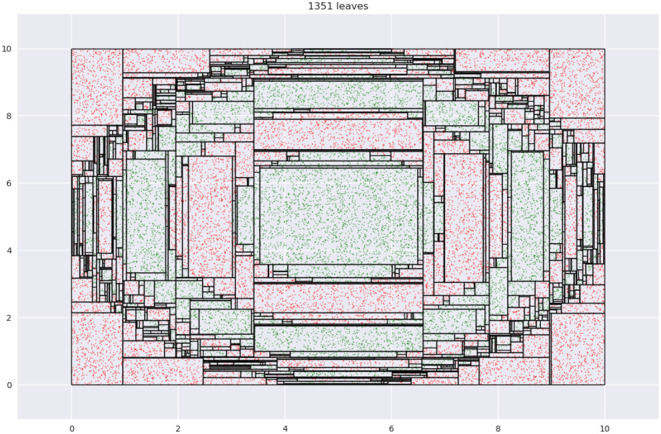
Tessellation of space produced by leaves of a decision tree.

Our key insight is an interesting property of decision trees (DT). A DT fragments its input space into axis-parallel rectangles. Figure 4 shows what this looks like when we learn a tree using CART on the dataset from Figure 1. Leaf regions are shown with the rectangles with the black edges.

**Figure 5:**
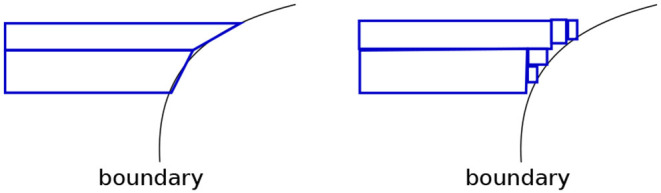
We see leaves of small areas because DTs are forced to approximate curvature with them.

Note how regions with relatively small areas almost always occur near boundaries. This happens here since none of the class boundaries are axis-parallel, and the DT, in being constrained in representation to axis-parallel rectangles, must use multiple small rectangles to approximate the curvature of the boundary. This is essentially *piecewise linear approximation* in high dimensions, with the additional constraint that the “linear pieces” be axis-parallel. Figure 5 shows a magnified view of the interaction of leaf edges with a curved boundary. The first panel shows how hypothetical trapezoid leaves might closely approximate boundary curvature. However, since the DT may only use axis-parallel rectangles, we are led to multiple small rectangles as an approximation, as shown in the second panel.

We exploit this geometrical property; in general, leaf regions with relatively small areas (volumes, in higher dimensions) produced by a DT, represent regions close to the boundary. Instead of directly determining an optimal *pdf* on the input space, we now do the following:

1. Learn a DT, with no size restrictions, on the data (*X*_*train*_, *Y*_*train*_). Assume the tree produces *m* leaves, where the region encompassed by a leaf is denoted by *R*_*i*_, 1 ≤ *i* ≤ *m*.
2. Define a *pmf* over the leaves, that assigns mass to a leaf in inverse proportion to its volume. Let *L* ∈ {1, 2, …, *m*} be a random variable denoting a leaf. Our *pmf* is $P_{L}\left(i\right)=P\left(L=i\right)=f\left(R_{i}\right),\text{ where }f\left(R_{i}\right)\propto vol{\left(R_{i}\right)}^{-1}$.
  The probability of sampling outside any *R*_*i*_ is set to 0.
3. To sample a point, sample a leaf first, based on the above *pmf*, and then sample a point from within this leaf assuming a uniform distribution:
  - Sample a leaf, *i* ~ *P*_*L*_.
  - Sample a point within this leaf, $x\sim U\left(R_{i}\right)$.
  - Since leaves are characterized by low entropy of the label distribution, we assign the majority label of leaf *i*, denoted by *label*(*i*), to the sampled point *x*.
    Assuming we have *k* unique labels, *label*(*i*) is calculated as follows:
    Let *S*_*i*_ = {*y*_*j*_:*y*_*j*_ ∈ *Y*_*train*_, *x*_*j*_ ∈ *X*_*train*_, *x*_*j*_ ∈ *R*_*i*_}. Then,
    (5) $$\begin{array}{r} label\left(i\right)=\underset{k}{\arg\;\max}{\hat{p}}_{ik} \end{array}$$
    (6) $$\begin{array}{rc} \text{where,} & {\hat{p}}_{ik}=\frac{1}{\left|S_{i}\right|}\underset{S_{i}}{\sum }I\left(y_{j}=k\right) \end{array}$$
    Note here that because of using $U\left(R_{i}\right)$ we may generate points *x* ∉ *X*_*train*_. Also, since a point *x* ∈ *R*_*i*_ ∩ *X*_*train*_ gets *assigned* *label*(*i*), the conditional distribution of labels approximately equals the original distribution:
    (7) $$\begin{array}{r} p\left(Y_{t}|X_{t}\right)\approx p\left(Y_{train}|X_{train}\right) \end{array}$$
    We call such a DT a *density tree*^6^ which we formally define as follows.

**Definition 3.1**. We refer to a DT as a **density tree** if (a) it is learned on (*X*_*train*_, *Y*_*train*_) with no size restrictions (b) there is a *pmf* defined over its leaves s.t. $P_{L}\left(i\right)=P\left(L=i\right)=f\left(R_{i}\right),\text{ where }f\left(R_{i}\right)\propto vol{\left(R_{i}\right)}^{-1}$.

Referring back to our desiderata, it should be clear how we address some of the challenges:

1. The location of class boundaries are naturally produced by DTs, in the form of (typically) low-volume leaf regions.
2. Instead of penalizing the lack of overlap with such boundary regions, we sample points in way that favors points close to class boundaries.
  Note that in relation to Equation (3) (reproduced below), *q* no longer ranges over all possible distributions; but over a restricted set relevant to the problem:
  (8) $$\begin{array}{r} p_{\eta }^{*}=\underset{q}{\arg\;\max}accuracy\left(train_{F}\left(q,\eta \right),p\right) \end{array}$$

We visit the issue of efficiency toward the end of this section.

**Figure 6:**
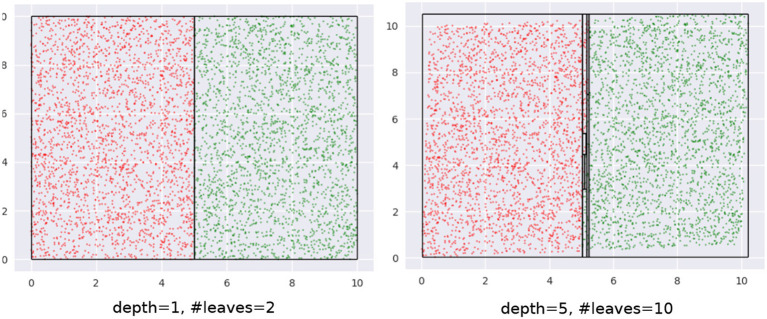
**(Left)** Axis parallel boundaries don't create small regions. **(Right)** This can be addressed by transforming the data. We see an increase in depth and the number of leaves of the density tree in the latter case.

**Figure 7:**
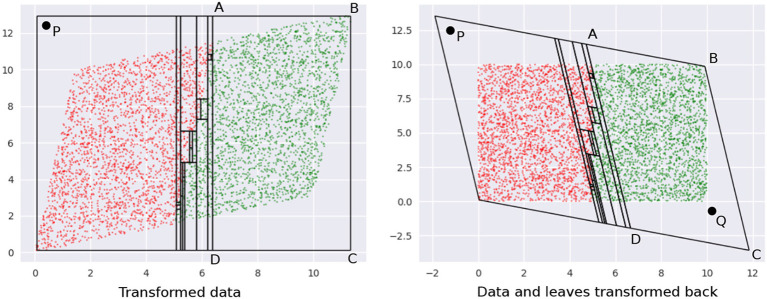
**(Left)** Transformed data. **(Right)** The leaves in the inverse transformation contain regions outside the bounding box of the original dataset. See text for a description of points P and Q.

**Figure 8:**
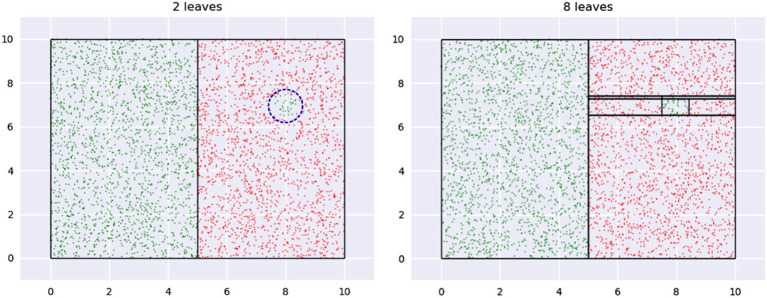
A region of low impact is shown in the first panel with a dashed blue circle. The first tree ignores this while a second, larger, tree creates a leaf for it.

**Figure 9:**
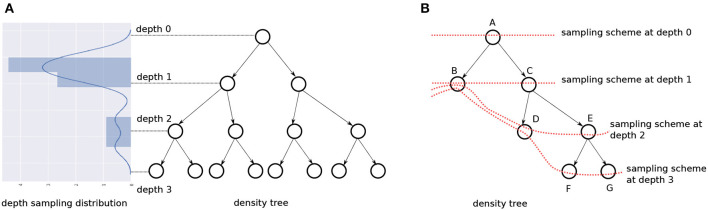
**(A)** The set of nodes at a depth have an associated *pmf* to sample from (not shown). A depth is picked based on the IBMM. **(B)** In case of an incomplete binary tree, we use the last available nodes closest to the depth being sampled from, so that the entire input space is represented. The red dotted lines show the nodes comprising the sampling scheme for different depths.

This simple scheme represents our approach at a high-level. However, this in itself is not sufficient to build a robust and efficient algorithm. We consider the following refinements to our approach:

1. ***pmf* at the leaf level**. What function *f* must we use to construct our *pmf* ? One could just use $f\left(R_{i}\right)=c\cdot vol{\left(R_{i}\right)}^{-1}$ where *c* is the normalization constant $c=1/\overset{m}{\underset{i=1}{\sum }}vol{\left(R_{i}\right)}^{-1}$. However, this quantity changes rapidly with volume. Consider a hypercube with edge-length *a* in *d* dimensions; the ratio of the (non-normalized) mass between this and another hypercube with edge-length *a*/2 is 2^*d*^. Not only is this change drastic, but it also has potential for numeric underflow.
  An alternative is to use a function that changes more slowly like the inverse of the length of the diagonal, $f\left(R_{i}\right)=c\cdot diag{\left(R_{i}\right)}^{-1}\text{ where }c=1/\overset{m}{\underset{i=1}{\sum }}diag{\left(R_{i}\right)}^{-1}$. Since DT leaves are axis-parallel hyperrectangles, *diag*(*R*_*i*_) is always well defined. In our hypercube example, the probability masses are $\propto 1/\left(a\sqrt{d}\right)$ and $\propto 1/\left(a\sqrt{d}/2\right)$ when the edge-lengths are *a* and *a*/2, respectively. The ratio of the non-normalized masses between the two cubes is now 2.
  This begs the question: is there yet another *pmf* we can use, that is optimal in some sense? Instead of looking for such an optimal *pmf*, we adopt the more pragmatic approach of starting with a “base” *pmf* —we use the inverse of the diagonal length—and then allowing the algorithm to modify it, via *smoothing*, to adapt it to the data.
2. **Smoothing**. Our algorithm may perform smoothing over the base *pmf* as part of the optimization. We use *Laplace smoothing* (Jurafsky and Martin, 2019, section 3.4), with λ as the smoothing coefficient. This modifies our *pmf* thus:
  (9) $$\begin{array}{r} f^{\prime }\left(R_{i}\right)=c(f\left(R_{i}\right)+\frac{\lambda }{m}) \end{array}$$
  Here, *c* is the normalization constant. The optimizer discovers the ideal value for λ.
  We pick Laplace smoothing because it is fast. Our framework, however, is general enough to admit a wide variety of options (discussed in section 5.2).
3. **Axis-aligned boundaries**. A shortcoming of our geometric view is if a boundary is axis-aligned, there are no leaf regions of small volumes along this boundary. This foils our sampling strategy. An easy way to address this problem is to transform the data by rotating or shearing it, and then construct a decision tree (see Figure 6). The image on the left shows a DT with two leaves constructed on the data that has an axis-parallel boundary. The image on the right shows multiple leaves around the boundary region, after the data is transformed (the transformation may be noticed at the top left and bottom right regions).
  The idea of transforming data by rotation is not new (Rodriguez et al., 2006; Blaser and Fryzlewicz, 2016). However, a couple of significant differences in our setup are:
  - We don't require rotation per se as our specific transformation; any transformation that produces small leaf regions near the boundary works for us.
  - Since *interpretability in the original input space* is our goal, we need to transform *back* our sample. This would not be required, say, if our only goal is to increase classification accuracy.
  The need to undo the transformation introduces an additional challenge: we cannot drastically transform the data since sampled points in the transformed space might be outliers in the original space. Figure 7 illustrates this idea, using the same data as in Figure 6.
  The first panel shows leaves learned on the data in the transformed space. Note how the overall region covered by the leaves is defined by the extremities—the top-right and bottom-left corners—of the region occupied by the transformed data. Any point within this rectangle is part of *some* leaf in a DT learned in this space. Consider point *P*—it is valid for our sampler to pick this. The second panel shows what the training data and leaf-regions look like when they are transformed back to the original space. Clearly, the leaves from the transformed space may not create a tight envelope around the data in the original space, and here, *P* becomes an outlier.
  Sampling a significant number of outliers is problematic because:
  - The validation and test sets do not have these points and hence learning a model on a training dataset with a lot of outliers would lead to sub-optimal accuracies.
  - There is no way to *selectively* ignore points like *P* in their leaf, since we uniformly sample within the entire leaf region. The only way to avoid sampling *P* is to ignore the leaf containing it (using an appropriate *pmf*); which is not desirable since it also forces us to ignore the non-outlier points within the leaf.
  Note that we also cannot transform the leaves back to the original space *first* and then sample from them, since (1) we lose the convenience and low runtime of uniform sampling $U\left(R_{i}\right)$: the leaves are not simple hyperrectangles any more; (2) for leaves not contained within the data bounding box in the original space, we cannot sample from the entire leaf region without risking obtaining outliers again–see point *Q* in $\bar{ABCD}$, in Figure 7.
  A simple and efficient solution to this problem is to only *slightly* transform the data, so that we obtain the small volume leaves at class boundaries (in the transformed space), but also, all valid samples are less likely to be outliers. This may be achieved by restricting the extent of transformation using a “near identity” matrix *A* ∈ ℝ^*d*×*d*^:
  (10) $$\begin{array}{r} {\left[A\right]}_{pq}=1,\text{if}p=q \end{array}$$
  (11) $$\begin{array}{rc} {\left[A\right]}_{pq} & \sim U\left(\left[0,\epsilon \right]\right),\text{if}p\neq q\text{, where}\epsilon \in {\mathbb{R}}_{>0}\text{is a small number.} \end{array}$$
  With this transformation, we would *still* be sampling outliers, but:
  - Their numbers are not significant now.
  - The outliers themselves are close to the data bounding box in the original space.
  These substantially weaken their negative impact on our technique.
  The tree is constructed on *AX*, where *X* is the original data, and samples from the leaves, $X_{t}^{\prime }$, are transformed back with $A^{-1}X_{t}^{\prime }$. Figure 6 is actually an example of such a near-identity transformation.
  A relevant question here is how do we know *when* to transform our data, i.e., when do we know we have axis-aligned boundaries? Since this is computationally expensive to determine, we always create multiple trees, each on a transformed version of the data (with different transformation matrices), and uniformly sample from the different trees. It is highly unlikely that *all* trees in this *bagging* step would have axis-aligned boundaries in their respective transformed spaces. Bagging also provides the additional benefit of low variance.
  We denote this bag of trees and their corresponding transformations by *B*. Algorithm 3 details how *B* is created. Our process is not too sensitive to the choice of epsilon, hence we set ϵ = 0.2 for our experiments.
4. **Selective Generalization**. Since we rely on geometric properties alone to define our *pmf*, all boundary regions receive a high probability mass irrespective of their contribution to classification accuracy. This is not desirable when the classifier is small and must focus on a few high impact regions. In other words, we prioritize all boundaries, but not all of them are valuable for classification; our algorithm needs a mechanism to ignore some of them. We refer to this desired ability of the algorithm as *selective generalization*.
  Figure 8 illustrates the problem and suggests a solution. The data shown has a small green region, shown with a dashed blue circle in the first panel, which we may want to ignore if we had to pick between learning its boundary or the relatively significant vertical boundary. The figure shows two trees of different depths learned on the data—leaf boundaries are indicated with solid black lines. A small tree, shown on the left, automatically ignores the circle boundary, while a larger tree, on the right, identifies leaves around it.
  Thus, one way to enable selective generalization is to allow our technique to pick a density tree of appropriate depth.
  But a shallow density tree is already part of a deeper density tree!–we can just sample at the depth we need. Instead of constructing density trees with different depths, we learn a “depth distribution” over fully grown density trees; drawing a sample from this tells us what fraction of the tree to consider.
  Figure 9A illustrates this idea. The depth distribution is visualized vertically and adjacent to a tree. We sample *r* ∈ [0, 1] from the distribution, and scale and discretize it to reflect a valid value for the depth. Let *depth*_*T*_() be the scaling/discretizing function for a tree *T*. Taking the tree in the figure as our example, *r* = 0 implies we sample our data instances from the nodes at *depth*_*T*_(*r*) = 0 i.e. at the *root*, and *r* = 0.5 implies we must sample from the nodes at *depth*_*T*_(*r*) = 1. We refer to the *pmf* for the nodes at a depth to be the *sampling scheme* at that depth. *T* has 4 sampling schemes—each capturing class boundary information at a different granularity, ranging from the root with no information and the leaves with the most information.
  We use an IBMM for the depth distribution. Similar to the one previously discussed in section 3.1.2, the depth-distribution has a parameter α for the DP and parameters {*a, b, a*′, *b*′} for its *Beta* priors. The significant difference is we have just one dimension now: the depth. The IBMM is shared across all trees in the bag; Algorithm 4 provides details at the end of this section.
5. **Revisiting label entropy**. When we sampled only from the leaves of a density tree, we could assign the majority label to the samples owing to the low label entropy. However, this is not true for nodes at intermediate levels—which the depth distribution might lead us to sample from. We deal with this change by defining an **entropy threshold** **E**. If the label distribution at a node has *entropy* ≤ *E*, we sample uniformly from the region encompassed by the node (which may be a leaf or an internal node) and use the majority label. However, if the *entropy* > *E*, we sample only among the *training* data instances that the node covers. Like ϵ, our technique is not very sensitive to a specific value of *E* (and therefore, need not be learned), as long as it is reasonably low: we use *E* = 0.15 in our experiments.
6. **Incomplete trees**. Since we use CART to learn our density trees, we have binary trees that are always *full*, but not necessarily *complete*, i.e., the nodes at a certain depth alone might not represent the entire input space. To sample at such depths, we “back up” to the nodes at the closest depth. Figure 9B shows this: at *depth* = 0 and *depth* = 1, we can construct our *pmf* with only nodes available at these depths, {*A*} and {*B, C*}, respectively, and still cover the whole input space. But for *depth* = 2 and *depth* = 3, we consider nodes {*B, D, E*} and {*B, D, F, G*}, respectively. The dotted red line connects the nodes that contribute to the sampling scheme for a certain depth.

Algorithm 4 shows how sampling from *B* works.

**Algorithm 3:**
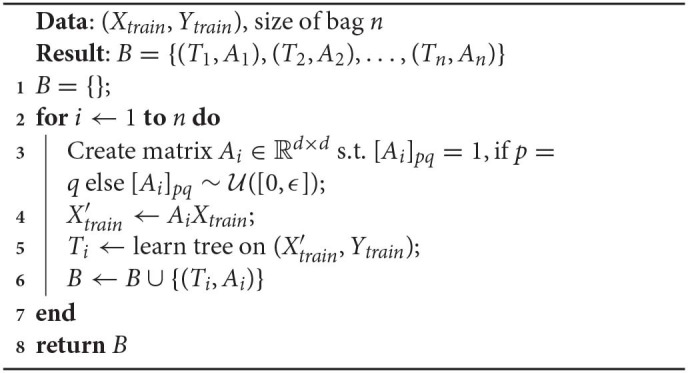
Create bag of density trees, *B*

**Algorithm 4:**
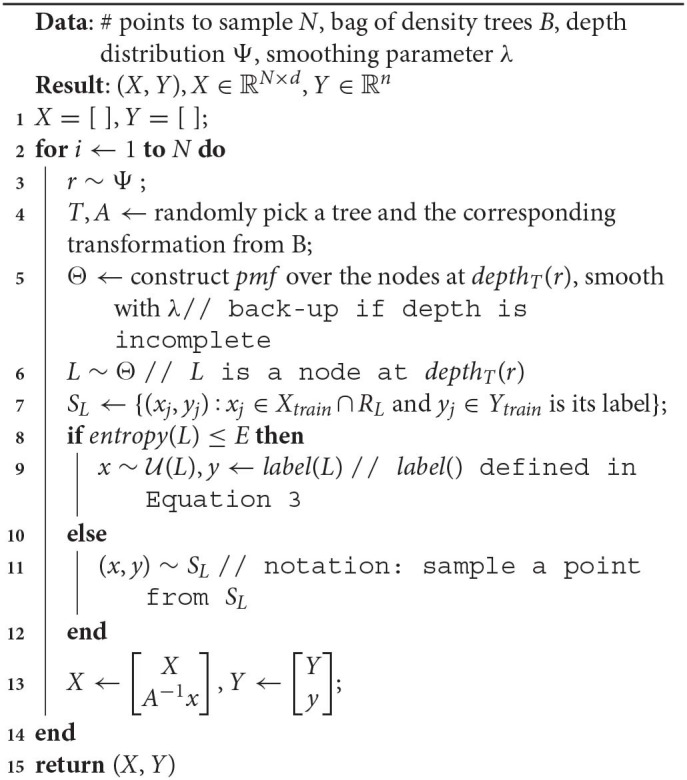
Sampling from a bag of density trees, *B*

     □

**Figure 10:**
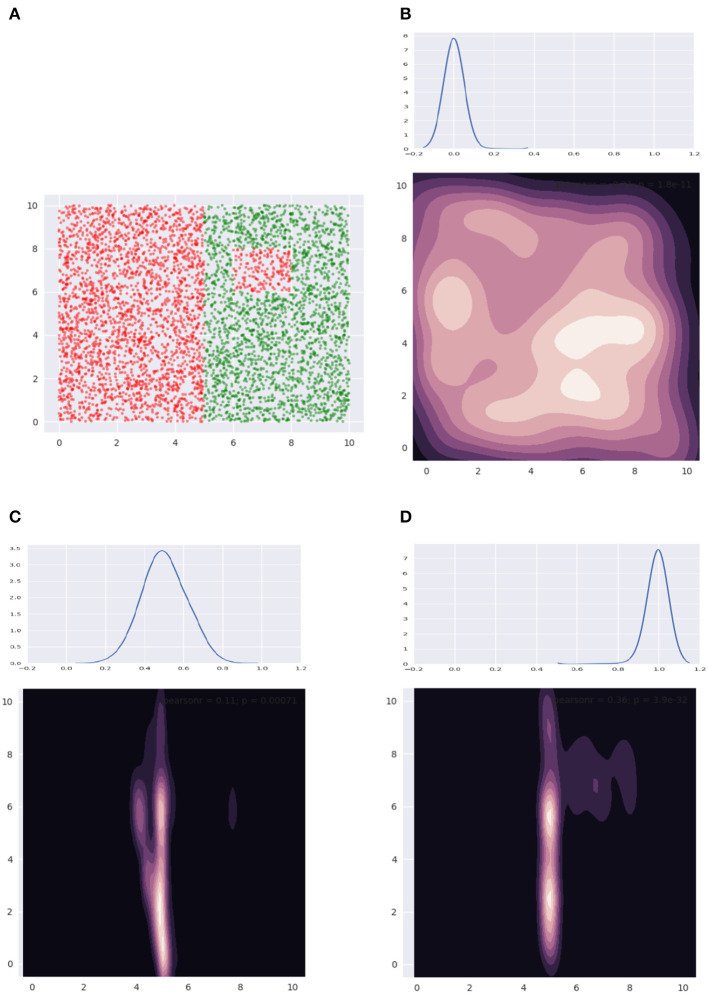
**(A)** Shows our dataset, while **(B–D)** show how the sample distribution varies with change of the depth distribution.

Figure 10 illustrates some of the distributions we obtain using our mechanism. Figure 10A shows our data—note, we only have axis-aligned boundaries. In Figures 10B–D, we show the depth distribution at the top, going from favoring the root in Figure 10B, to nodes halfway along the height of the tree in Figure 10C, finally to the leaves in Figure 10D. The contour plot visualizes the distributions, where a lighter color indicates relatively higher sample density. We see that in Figure 10B, we sample everywhere in the data bounding box. In Figure 10C, the larger boundary is identified. In Figure 10D, the smaller boundary is also identified. A bag of size 5 was used and the smoothing coefficient λ was held constant at a small value.

This completes the discussion of the salient details of our sampling technique. The optimization variables are summarized below:

1. λ, the Laplace smoothing coefficient.
2. α, the DP parameter.
3. {*a, b, a*′, *b*′}, the parameters of the *Beta* priors for the IBMM depth distribution. A component/partition *i* is characterized by the distribution *Beta*(*A*_*i*_, *B*_*i*_), where *A*_*i*_ ~ *Beta*(*a, b*), $B_{i}\sim Beta\left(a^{\prime },b^{\prime }\right)$.
  The IBMM and its parameters, {α, *a, b, a*′, *b*′}, are shared across all trees in the bag *B*, and λ is shared across all sampling schemes.
  We also introduced two additional parameters: ϵ and *E*. As mentioned previously, we do not include them in our optimization since our process is largely insensitive to their precise values as long as these are reasonably small. We use ϵ = 0.2 and *E* = 0.15 for our experiments.
  The above parameters exclusively determine how the sampler works. In addition, we propose the following parameters:
4. *N*_*s*_ ∈ ℕ, sample size. The sample size can have a significant effect on model performance. We let the optimizer determine the best sample size to learn from. We constrain *N*_*s*_ to be larger than the minimum number of points needed for statistically significant results.
  Note that we can allow *N*_*s*_ > |*X*_*train*_|. This larger sample will be created by either repeatedly sampling points - at nodes where the label *entropy* > *E*—or by generating synthetic points, when *entropy* ≤ *E*.
5. *p*_*o*_ ∈ [0, 1]—proportion of the sample from the original distribution. Given a value for *N*_*s*_, we sample (1−*p*_*o*_)*N*_*s*_ points from the density tree(s) and *p*_*o*_*N*_*s*_ points (stratified) from our training data (*X*_*train*_, *Y*_*train*_).
  Recall that our hypothesis is that learning a distribution helps until a size η′ (Equation 3). Beyond this size, we need to provide a way for the sampler to reproduce the original distribution. While it is possible the optimizer finds a Ψ_*t*_ that corresponds to this distribution, we want to make this easier: now the optimizer can simply set *p*_*o*_ = 1. Essentially, *p*_*o*_ is way to “short-circuit” the discovery of the original distribution.
  This variable provides the additional benefit that observing a transition *p*_*o*_ = 0 → 1, as the model size increases, would empirically validate our hypothesis.

We have a total of **eight optimization variables** in this technique. The variables that influence the sampling behavior are collectively denoted by Ψ = {α, *a, b, a*′, *b*′}. The complete set of variables is denoted by Φ = {Ψ, *N*_*s*_, λ, *p*_*o*_}.

This is a welcome departure from our naive solution: the number of optimization variables does not depend on the dimensionality *d* at all! Creating density trees as a preprocessing step gives us a *fixed set of 8 optimization variables* for any data. This makes the algorithm much more efficient than before, and makes it practical to use for real world data.

Algorithm 5 shows how we modify our naive solution to incorporate the new sampler.

**Algorithm 5:**
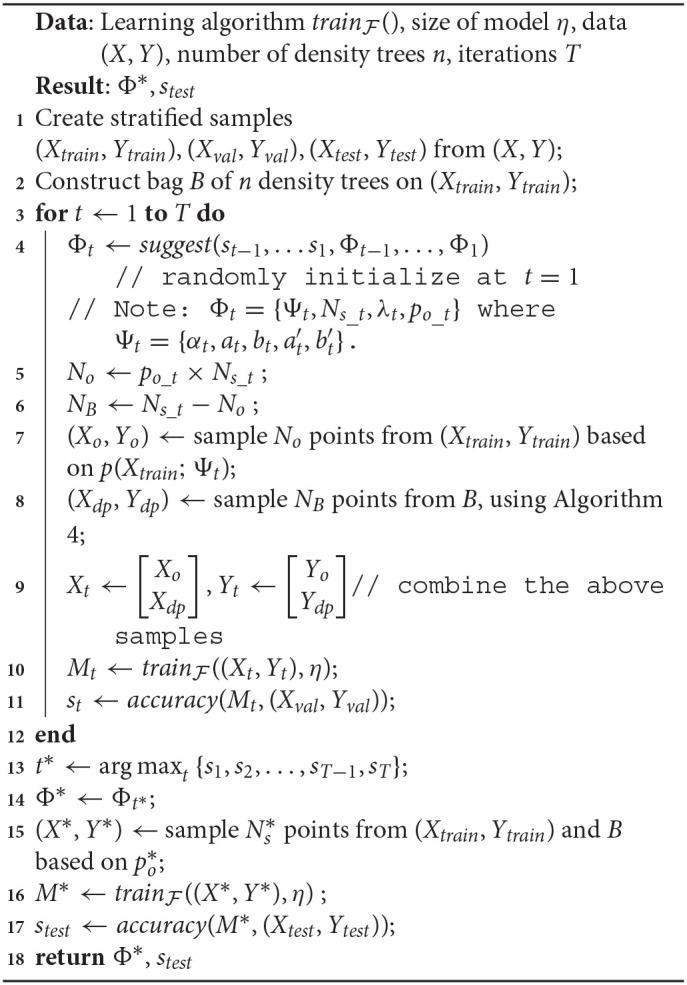
Adaptive sampling using density trees

As before, we discover the optimal Φ using TPE as the optimizer and *accuracy*() as the fitness function. We begin by constructing our bag of density trees, *B*, on transformed versions of (*X*_*train*_, *Y*_*train*_), as described in Algorithm 3. At each iteration in the optimization, based on the current value *p*_*o*_*t*_, we sample data from *B* and (*X*_*train*_, *Y*_*train*_), train our model on it, and evaluate it on (*X*_*val*_, *Y*_*val*_). In our implementation, lines 7–11 are repeated (thrice, in our experiments) and the accuracies are averaged to obtain a stable estimate for *s*_*t*_.

Additional details pertaining to Algorithm 5:

1. We use a *train* : *val* : *test* split ratio of 60 : 20 : 20.
2. The training step to build model *M*_*t*_ in line 10, takes into account class imbalance: it either balances the data by sampling (this is the case with a *Linear Probability Model*), or it uses an appropriate cost function or instance weighting, to simulate balanced classes (this is case with DTs or *Gradient Boosted Models*).
  However, it is important to note that both (*X*_*val*_, *Y*_*val*_)and(*X*_*test*_, *Y*_*test*_) represent the original distribution, and thus indeed test the efficacy of our technique on data with varying degrees of class imbalance.

## 4. Experiments

This section discusses experiments that validate our technique and demonstrate its practical utility. We describe our experimental setup in section 4.1 and present our observations and analysis in section 4.2.

### 4.1. Setup

We evaluate Algorithm 5 using 3 different learning algorithms, i.e., $train_{F}\left(\;\right)$, on 13 real world datasets. We construct models for a wide range of sizes, η, to comprehensively understand the behavior of the algorithm. For each combination of dataset, learning algorithm and model size, we record the percentage *relative improvement* in the *F*1(macro) score on (*X*_*test*_, *Y*_*test*_) compared to the *baseline* of training the model on the original distribution:

$$\begin{array}{l} \delta F1=\frac{100\times \left(F1_{new}-F1_{baseline}\right)}{F1_{baseline}} \end{array}$$

We specifically choose the *F*1 *macro* metric as it accounts for class imbalance, e.g., it penalizes the score even if the model performs well on a majority class but poorly on a minority class.

Since the original distribution is part of the optimization search space, i.e., when *p*_*o*_ = 1, the lowest improvement we report is 0%, i.e., δ*F*1 ∈ [0, ∞). All reported values of δ*F*1 are averaged over **five** runs of Algorithm 5. As mentioned before, in *each* such run, lines 7–11 in the algorithm are repeated thrice to obtain a robust estimate for *accuracy*(), and thus, *s*_*t*_.

We also perform *upper-tailed paired sample t-tests*, with a *p_value* threshold of 0.1, to assess if the mean of the *F*1_*new*_ scores are higher than the mean of the *F*1_*baseline*_ scores, in a statistically significant way.

#### 4.1.1. Data

We use a variety of real-world datasets, with different dimensionalities, number of classes and different class distributions to test the generality of our approach. The datasets were obtained from the LIBSVM website (Chang and Lin, 2011), and are described in Table 1. The column “Label Entropy,” quantifies the extent of class imbalance, and is computed for a dataset with *C* classes in the following way:

(12) $$\begin{array}{r} \text{Label Entropy}=\underset{j\in \left\{1,2,\ldots ,C\right\}}{\sum }-p_{j}\log_{C}p_{j}\\ \text{Here,}p_{j}=\frac{\left|\left\{x_{i}|y_{i}=j\right\}\right|}{N} \end{array}$$

Values close to 1 imply classes are nearly balanced in the dataset, while values close to 0 represent relative imbalance.

**Table 1:** Datasets: we use the dataset versions available on the LIBSVM website (Chang and Lin, 2011). However, we have mentioned the original source in the “Description” column.

**S. No**.	**Dataset**	**Dimensions**	**# Classes**	**Label entropy**	**Description**
1	cod-rna	8	2	0.92	Predict presence of non-coding RNA common to a pair of RNA sequences, based on individual sequence properties and their similarity (Uzilov et al., 2006).
2	ijcnn1	22	2	0.46	Time series data produced by an internal combustion engine is used to predict normal engine firings vs. misfirings (Prokhorov, 2001). Transformations as in Chang and Lin (2001).
3	higgs	28	2	1.00	Predict if a particle collision produces Higgs bosons or not, based on collision properties (Baldi et al., 2014).
4	covtype.binary	54	2	1.00	Modification of the *covtype* dataset (see row 12), where classes are divided into two groups (Collobert et al., 2002).
5	phishing	68	2	0.99	Various website features are used to predict if the website is a *phishing* website (Mohammad et al., 2012). Transformations used as in Juan et al. (2016)
6	a1a	123	2	0.80	Predict whether a person makes over 50K a year, based on census data variables (Dua and Graff, 2017). Transformations as in Platt (1998).
7	pendigits	16	10	1.00	Classify handwritten digit samples into the digits 0–9 (Alimoglu and Alpaydin, 1996; Dua and Graff, 2017).
8	letter	16	26	1.00	Images of the capital letters A–Z were produced by random distortion of these characters from 20 fonts. The task is to classify these character images as one of the original letters (Michie et al., 1995). Transformations as in Hsu and Lin (2002).
9	Sensorless	48	11	1.00	Based on phase current measurements of an electric motor, predict different error conditions (Paschke et al., 2013). We use the transformations from Wang et al. (2018).
10	senseit_aco	50	3	0.95	Predict vehicle type using acoustic data gathered by a sensor network (Duarte and Hu, 2004).
11	senseit_sei	50	3	0.94	Predict vehicle type using seismic data gathered by a sensor network (Duarte and Hu, 2004).
12	covtype	54	7	0.62	Predicting forest cover type from cartographic variables (Dean and Blackard, 1998; Dua and Graff, 2017).
13	connect-4	126	3	0.77	Predict if the first player wins, loses or draws, based on board positions of the board game *Connect Four* (Dua and Graff, 2017).

#### 4.1.2. Models

We use the following model families, F, and learning algorithms, $train_{F}\left(\;\right)$, in our experiments:

1. *Decision Trees*: We use the implementation of CART in the *scikit-learn* library (Pedregosa et al., 2011). Our notion of size here is the depth of the tree.
  *Sizes*: For a dataset, we first learn an optimal tree *T*_*opt*_ based on the *F1-score*, without any size constraints. Denote the depth of this tree by *depth*(*T*_*opt*_). We then try our algorithm for these settings of CART's *max*_*depth* parameter: {1, 2, …, *min*(*depth*(*T*_*opt*_), 15)}, i.e., we experiment only up to a model size of 15, stopping early if we encounter the optimal tree size. Stopping early makes sense since the model has attained the size needed to capture all patterns in the data; changing the input distribution is not going to help beyond this point.
  Note that while our notion of size is the *actual* depth of the tree produced, the parameter we vary is *max*_*depth*; this is because decision tree libraries do not allow specification of an exact tree depth. This is important to remember since CART produces trees with actual depth up to as large as the specified *max*_*depth*, and therefore, we might not see actual tree depths take all values in {1, 2, …, *min*(*depth*(*T*_*opt*_), 15)}, e.g., *max*_*depth* = 5 might give us a tree with *depth* = 5, *max*_*depth* = 6 might also result in a tree with *depth* = 5, but *max*_*depth* = 7 might give us a tree with *depth* = 7. We report relative improvements at actual depths.
2. *Linear Probability Model (LPM)* (Mood, 2010): This is a linear classifier. Our notion of size is the number of terms in the model, i.e., features from the original data with non-zero coefficients. We use our own implementation based on *scikit-learn*. Since LPMs inherently handle only binary class data, for a multiclass problem, we construct a *one-vs-rest* model, comprising of as many binary classifiers as there are distinct labels. The given size is enforced for *each* binary classifier. For instance, if we have a 3-class problem, and we specify a size of 10, then we construct 3 binary classifiers, each with 10 terms. We did not use the more common *Logistic Regression* classifier because: (1) from the perspective of interpretability, LPMs provide a better sense of variable importance (Mood, 2010) (2) we believe our effect is equally well illustrated by either linear classifier.
  We use the *Least Angle Regression* (Efron et al., 2004) algorithm, that grows the model one term at a time, to enforce the size constraint.
  *Sizes*: For a dataset with dimensionality *d*, we construct models of sizes: {1, 2, …, *min*(*d*, 15)}. Here, the early stopping for LPM happens only for the dataset *cod-rna*, which has *d* = 8. All other datasets have *d* > 15 (see Table 1).
3. *Gradient Boosted Model (GBM)*: We use decision trees as our base classifier in the boosting. Our notion of size is the number of trees in the boosted forest for a *fixed maximum depth* of the base classifiers. We use the *LightGBM* library (Ke et al., 2017) for our experiments.
  We run two sets of experiments with the GBM, with maximum depths fixed at 2 and 5. This helps us compare the impact of our technique when the model family F inherently differs in its effective capacity, e.g., we would expect a GBM with 10 trees and a maximum depth of 5 to be more accurate than a GBM with 10 trees and a maximum depth of 2.
  *Sizes*: If the optimal number of boosting rounds for a dataset is *r*_*opt*_, we explore the model size range: {1, 2, …, *min*(*r*_*opt*_, 10)}. We run two sets of experiments with GBM—one using base classification trees with *max*_*depth* = 2, and another with *max*_*depth* = 5. Both experiments use the same range for size/boosting rounds.

The density trees themselves use the CART implementation in *scikit-learn*. We use the *Beta* distribution implementation provided by the *SciPy* package (Jones et al., 2001).

#### 4.1.3. Parameter Settings

Since TPE performs optimization with *box constraints*, we need to specify our search space for the various parameters in Algorithm 5:

1. λ: this is varied in the *log-space* such that log_10_λ ∈ [−3, 3].
2. *p*_*o*_: We want to allow the algorithm to arbitrarily mix samples from *B* and (*X*_*train*_, *Y*_*train*_). Hence, we set *p*_*o*_ ∈ [0, 1].
3. *N*_*s*_: We set *N*_*s*_ ∈ [1000, 10000]. The lower bound ensures that we have statistically significant results. The upper bound is set to a reasonably large value.
4. α: For a DP, α ∈ ℝ_>0_. We use a lower bound of 0.1.
  We rely on the general properties of a DP to estimate an upper bound, α_*max*_. Given α, for *N* points, the expected number of components *k* is given by:
  (13) $$\begin{array}{r} E\left[k|\alpha \right]=O\left(\alpha H_{N}\right) \end{array}$$
  (14) $$\begin{array}{r} E\left[k|\alpha \right]\leq \alpha H_{N} \end{array}$$
  (15) $$\begin{array}{r} \alpha \geq \frac{E\left[k|\alpha \right]}{H_{N}} \end{array}$$
  Here, *H*_*N*_ is the *N*th *harmonic sum* (see Blei, 2007).
  Since our distribution is over the depth of a density tree, we already know the maximum number of components possible, *k*_*max*_ = 1+depth of density tree. We use *N* = 1, 000, since this is the lower bound of *N*_*s*_, and we are interested in the upper bound of α (note *H*_*N*_ ∝ *N*—see section 1.3). We set *k*_*max*_ = 100 (this is greater than any of the density tree depths in our experiments) to obtain a liberal upper bound, α_*max*_ = 100/*H*_1000_ = 13.4. Rounding up, we set α ∈ [0.1, 14]^7^.
  We draw a sample from the IBMM using *Blackwell-MacQueen* sampling (Blackwell and MacQueen, 1973).
5. {*a, b, a*′, *b*′}: Each of these parameters are allowed a range [0.1, 10] to admit various shapes for the *Beta* distributions.

We need to provide a budget *T* of iterations for the TPE to run. In the case of DT, GBM and binary class problems for LPM, *T* = 3, 000. Since multiclass problems in LPM require learning multiple classifiers, leading to high running times, we use a lower value of *T* = 1, 000. We arrived at these budget values by trial and error; not low enough to lead to inconclusive results, not unreasonably high to run our experiments.

### 4.2. Observations and Analysis

We break down our results and discussion by the $train_{F}\left(\;\right)$ used.

#### 4.2.1. DT Results

The DT results are shown in Table 2. A series of unavailable scores, denoted by “-,” toward the right end of the table for a dataset denotes we have already reached its optimal size. For example, in Table 2, *cod-rna* has an optimal size of 10.

**Table 2:** Classification Results with DTs.

**Depth =**	**1**	**2**	**3**	**4**	**5**	**6**	**7**	**8**	**9**	**10**	**11**	**12**	**13**	**14**	**15**
**Datasets**															
cod-rna	4.55	0.82	**0.24**	**2.18**	**0.29**	0.12	0.22	**0.22**	**0.17**	0.00	–	–	–	–	–
ijcnn1	**3.20**	12.96	**8.65**	**9.04**	**3.38**	**1.20**	**2.36**	**1.22**	**0.93**	0.00	**0.25**	**0.39**	**0.60**	0.00	0.68
higgs	3.75	**0.94**	**0.94**	0.45	0.00	0.47	–	–	–	–	–	–	–	–	–
covtype.binary	0.41	0.33	**0.79**	**0.78**	**0.76**	**0.76**	0.41	0.57	0.63	**0.82**	0.00	1.64	–	–	–
phishing	**0.00**	0.70	0.29	**0.24**	**0.62**	**0.11**	**0.11**	**0.35**	0.07	0.10	0.00	0.00	0.00	0.00	0.00
a1a	0.00	**5.09**	5.60	**4.77**	**4.09**	**2.40**	**3.45**	**0.82**	**1.83**	-	**0.65**	0.00	2.35	–	0.92
pendigits	10.49	**3.08**	**4.46**	**9.81**	**4.66**	**2.18**	**1.04**	**0.07**	0.09	0.00	0.00	0.00	0.00	0.00	0.10
letter	1.04	**10.52**	**32.46**	46.79	**45.96**	**20.70**	**9.35**	**5.05**	**4.21**	**2.14**	**0.54**	0.00	0.00	0.00	0.00
Sensorless	0.00	**41.97**	**71.69**	81.43	**38.33**	**16.76**	**7.86**	**4.30**	**2.20**	**1.10**	**0.63**	0.16	0.75	0.28	0.00
senseit_aco	19.64	**0.57**	**3.09**	**1.47**	**1.49**	**0.47**	0.39	0.93	–	–	–	–	–	–	–
senseit_sei	2.18	**1.04**	**2.15**	0.53	**0.97**	**1.02**	0.43	–	–	–	–	–	–	–	–
covtype	**27.80**	101.80	**18.62**	**7.01**	**6.30**	**2.41**	**2.11**	**2.40**	1.64	2.01	2.58	**1.82**	0.00	**0.69**	0.52
connect-4	181.33	**32.51**	**18.12**	**12.78**	**7.33**	**11.14**	**4.19**	**6.10**	**2.71**	1.19	**2.78**	**2.08**	2.13	2.08	**1.55**

*Values indicate improvements δF1, averaged over five runs. Entries in bold are statistically significant. Underlined entries denote the best improvement for a dataset*.

For each dataset, the best improvement across different sizes is shown underlined. As mentioned before, we perform *upper-tailed paired sample t-tests* at a *p*-value threshold of 0.1, to compare the original and new *F*1 scores. Table 2 shows the statistically significant entries in bold, and entries that are not statistically significant are shown in regular font. The horizontal line separates binary datasets from multiclass datasets.

**Figure 11:**
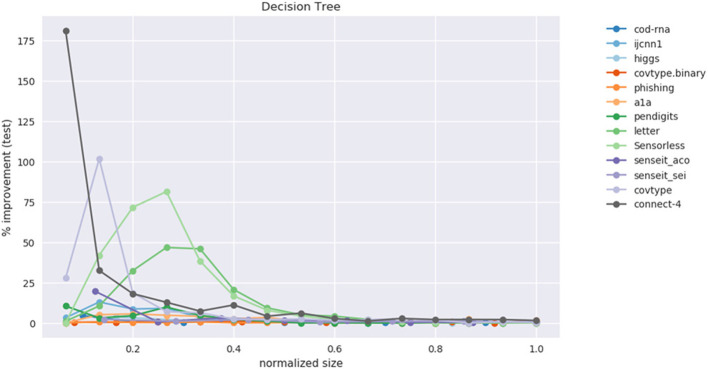
Improvement in F1 score on test with increasing size. Data in Table 2.

This data is also visualized in Figure 11. The x-axis shows a scaled version of the actual tree depths for easy comparison: if the largest actual tree depth explored is η_*max*_ for a dataset, then a size η is represented by η/η_*max*_. This allows us to compare a dataset like *cod-rna*, which only has models up to a size of 10, with *covtype*, where model sizes go all the way up to 15.

We observe significant improvements in the F1-score for at least one model size for majority of the datasets. The best improvements themselves vary a lot, ranging from 0.70% for *phishing* to 181.33% for *connect-4*. More so, these improvements seem to happen at small sizes: only one best score—for *covtype.binary*—shows up on the right half of Table 2. This is inline with Equations (3) and (4): beyond a model size η′, δ*F*1 = 0%.

**Figure 12:**
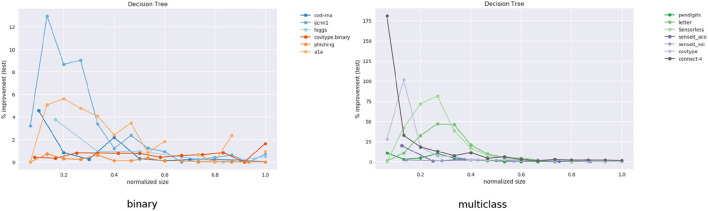
Performance on binary vs. multi-class classification problems using CART. This is an elaboration of Figure 11.

It also seems that we do much better with multiclass data than with binary classes. Because of the large variance in improvements, this is hard to observe in Figure 11. However, if we separate the binary and multiclass results, as in Figure 12, we note that there are improvements in both the binary and multiclass cases, and the magnitude in the latter are typically higher (note the y-axes). We surmise this happens because, in general, DTs of a fixed depth have a harder problem to solve when the data is multiclass, providing our algorithm with an easier baseline to beat.

Class imbalance itself doesn't seem to play a role. As per Table 1, the datasets with most imbalance are *ijcnn1, covtype, connect-4*, for which we see best improvements of 12.96, 101.80, 181.33%, respectively.

Most of the statistically significant results occur at small model sizes (note how most bold entries are on the left half of the table), reinforcing the validity of our technique. Since some of the models go up to (or close to) the optimal model size—the last column in Table 2 for these datasets are either empty or tending to 0 (all datasets except *ijcnn1, a1a, covtype, connect-4* satisfy this condition)—a significant δ*F*1 is also not expected.

**Figure 13:**
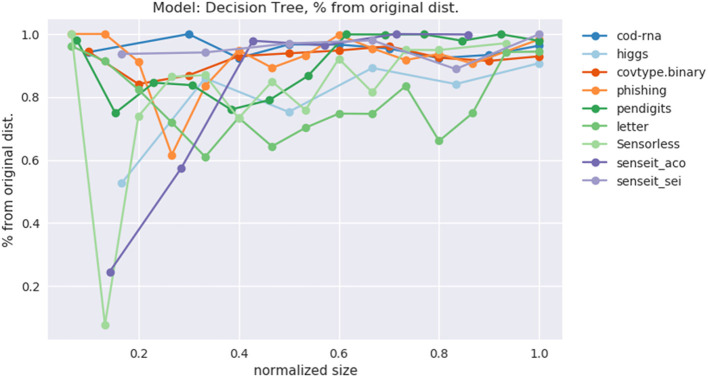
Variation of *p*_*o*_ with increasing model size.

Figure 13 shows the behavior of *p*_*o*_, *only* for the datasets where our models have grown close to the optimal size. Thus, we exclude *ijcnn1, a1a, covtype, connect-4*. We observe that indeed *p*_*o*_ → 1 as our model grows to the optimal size. This empirically validates our hypothesis from section 2.1, that smaller models prefer a distribution different from the original distribution to learn from, but the latter is optimal for larger models. And we gradually transition to it as model size increases.

Demonstrating this effect is a key contribution of our work.

We are also interested in knowing what the depth-distribution IBMM looks like. This is challenging to visualize for multiple datasets in one plot, since we have an optimal IBMM learned by our optimizer, for *each* model size setting. We summarize this information for a dataset in the following manner:

1. Pick a sample size of *N* points to use.
2. We allocate points to sample from the IBMM for a particular model size, in proportion of δ*F*1. For instance, if we have experimented with three model sizes, and δ*F*1 are 7, 11, and 2%, we sample 0.35, 0.55, and 0.1*N* points, respectively from the corresponding IBMMs.
3. We fit a *Kernel Density Estimator (KDE)* over these *N* points, and plot the KDE curve. This plot represents the IBMM across model sizes for a dataset *weighted* by the improvement seen for a size.

*N* should be large enough that the visualization is robust to sample variances. We use *N* = 10, 000.

**Figure 14:**
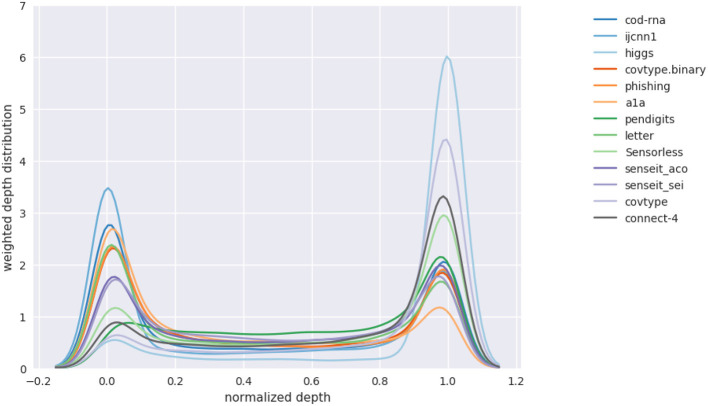
Distribution over levels in density tree(s). Aggregate of distribution over different model sizes.

Figure 14 shows such a plot for DTs. The x-axis represents the depth of the density tree normalized to [0, 1]. The smoothing by the KDE causes some spillover beyond these bounds.

We observe that, in general, the depth distribution is concentrated either near the root of a density tree, where we have little or no information about class boundaries and the distribution is nearly identical to the original distribution, or at the leaves, where we have complete information of the class boundaries. An intermediate depth is relatively less used. This pattern in the depth distribution is *surprisingly consistent* across all the models and datasets we have experimented with. We hypothesize this might be because of the following reasons:

1. The information provided at an intermediate depth—where we have moved away from the original distribution, but have not yet completely discovered the class boundaries—might be relatively noisy to be useful.
2. The model can selectively generalize well enough from the complete class boundary information at the leaves.

Note that while fewer samples are drawn at intermediate depths, the number is not always insignificant—as an example, see *pendigits* in Figure 14; hence using a distribution across the height of the density tree is still a useful strategy.

#### 4.2.2. LPM Results

The results for LPM are shown in Table 3. The improvements look different from what we observed for DT, which is to be expected across different model families. Notably, compared to DTs, there is no prominent disparity in the improvements between binary class and multiclass datasets. Since the LPM builds *one-vs.-rest* binary classifiers in the multiclass case, and the size restriction—number of terms—applies to each individually, this intuitively makes sense. This is unlike DTs where the size constraint was applied to a single multiclass classifier. However, much like DTs, we still observe the pattern of the greatest improvements occurring at relatively smaller model sizes.

**Table 3:** Classification Results with LPMs.

**# Terms =**	**1**	**2**	**3**	**4**	**5**	**6**	**7**	**8**	**9**	**10**	**11**	**12**	**13**	**14**	**15**
**Datasets**															
cod-rna	**22.10**	**28.82**	**51.12**	**56.93**	59.33	**48.89**	**24.52**	**6.75**	–	–	–	–	–	–	–
ijcnn1	17.90	**8.20**	**1.05**	**1.64**	**1.88**	**1.28**	**1.25**	**2.03**	**2.30**	**1.73**	**1.06**	**2.27**	**1.54**	**0.92**	**2.77**
higgs	0.00	0.02	**1.34**	**1.98**	**3.01**	**3.95**	**4.58**	**3.80**	**4.92**	**5.77**	6.89	**6.26**	**5.49**	**4.73**	**4.67**
covtype.binary	0.08	**1.91**	**2.94**	**5.57**	**8.47**	**11.53**	**13.55**	**7.24**	**12.00**	**11.59**	**13.41**	13.72	**11.73**	**11.99**	**11.23**
phishing	0.00	0.00	0.00	0.00	**0.12**	**0.17**	**0.59**	0.03	0.07	0.21	0.15	0.27	**0.41**	**0.56**	0.66
a1a	0.00	**20.93**	48.37	**37.92**	**29.01**	**18.84**	**7.93**	8.89	**9.44**	**5.68**	**3.78**	**3.39**	**3.29**	**1.88**	**1.73**
pendigits	**8.52**	**9.33**	9.87	**6.97**	**8.74**	**4.01**	**4.16**	**0.91**	**1.13**	**0.47**	**0.49**	**0.28**	**1.00**	**0.51**	**0.59**
letter	**12.00**	**9.27**	19.13	**10.41**	**10.62**	**3.30**	**2.68**	**2.21**	**2.08**	**2.94**	**2.48**	**2.24**	**3.63**	**4.94**	**4.84**
Sensorless	72.16	**56.25**	**33.11**	**12.04**	**14.60**	**15.27**	**17.34**	**24.99**	**22.80**	**26.49**	**29.54**	**33.55**	**43.44**	**43.24**	**43.22**
senseit_aco	4.51	59.50	**33.34**	**19.42**	**17.09**	**11.26**	**11.90**	**8.25**	**5.67**	**4.39**	**3.53**	**2.79**	**2.83**	**1.40**	**1.79**
senseit_sei	147.09	**46.13**	**19.03**	**7.70**	**2.53**	**0.96**	**1.10**	**1.38**	**0.95**	**1.26**	**1.11**	**1.36**	**1.33**	**0.31**	**0.45**
covtype	27.84	**20.15**	**6.25**	**4.98**	**2.29**	**6.90**	**2.76**	**5.02**	**7.67**	**7.20**	**7.06**	**8.18**	**7.78**	**9.21**	**8.84**
connect-4	76.68	**20.12**	**20.62**	**12.12**	**6.25**	**3.84**	**4.84**	**4.03**	**4.64**	**3.52**	**3.20**	**1.87**	**1.93**	0.67	**2.38**

*Values indicate improvements δF1, averaged over five runs. Entries in bold are statistically significant. Underlined entries denote the best improvement for a dataset*.

**Figure 15:**
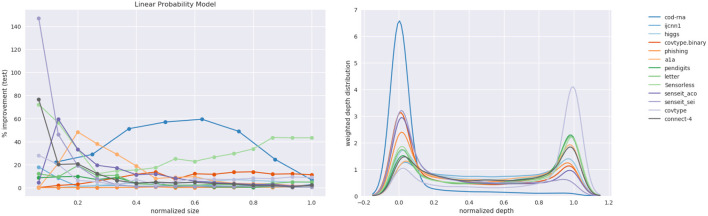
Linear Probability Model: improvements and the distribution over depths of the density trees.

Figure 15 shows the plots for improvement in the F1-score and the weighted depth distribution. The depth distribution plot displays concentration near the root and the leaves, similar to the case of the DT in Figure 14.

Note that unlike the case of the DT, we haven't determined how many terms the optimal model for a dataset has; we explore up to *min*(*d*, 15). Nevertheless, as in the case of DTs, we note the pattern that the best improvements typically occur at smaller sizes: only *higgs* exhibits its largest improvements at a relatively large model size in Table 3. Here too, class imbalance doesn't seem to play a role (datasets with most imbalance—*ijcnn1, covtype, connect-4*—show best improvements of 17.9, 27.84, 76.68%, respectively, and most results at small model sizes are statistically significant.

#### 4.2.3. GBM Results

An interesting question to ask is how, if at all, the *bias* of the model family of F in Algorithm 5, influences the improvements in accuracy. We cannot directly compare DTs with LPMs since we don't know how to order models from different families: we cannot decide how large a DT to compare to a LPM with, say, 4 non-zero terms.

To answer this question we look at GBMs where we identify two levers to control the model size. We consider two different GBM models—with the *max*_*depth* of base classifier trees as 2 and 5, respectively. The number of boosting rounds is taken as the size of the classifier and is varied from 1 to 10. We refer to the GBMs with base classifiers with *max*_*depth* = 2 and *max*_*depth* = 5 as representing weak and strong model families, respectively.

We recognize that qualitatively there are two opposing factors at play:

1. A weak model family implies it might not learn sufficiently well from the samples our technique produces. Hence, we expect to see smaller improvements than when using a stronger model family.
2. A weak model family implies there is a lower baseline to beat. Hence, we expect to see larger improvements.

We present an abridged version of the GBM results in Table 4 in the interest of space. The complete results are made available in Table A1 in the Appendix. We present both the improvement in the *F*1 score, δ*F*1, and its new value, *F*1_*new*_.

**Table 4:** Classification Results with GBMs.

		**Boosting rounds =**	**1**	**2**	**3**	**4**	**5**	**6**	**7**	**8**	**9**	**10**
**Datasets**	**Max depth**	**Score type**										
Sensorless	2	*F*1	0.76	0.77	0.78	0.80	0.80	0.80	0.81	0.81	0.81	0.81
		δ*F*1	3.19	3.36	3.11	5.05	4.13	1.75	3.22	1.98	1.90	2.43
	5	*F*1	0.91	0.92	0.93	0.94	0.94	0.94	0.94	0.95	0.95	0.95
		δ*F*1	0.29	0.26	0.16	0.41	0.00	0.18	0.37	0.30	0.00	0.26
senseit_aco	2	*F*1	0.22	0.24	0.31	0.37	0.52	0.59	0.61	0.62	0.63	0.63
		δ*F*1	0.00	6.81	41.41	67.44	88.57	9.76	6.97	4.73	2.34	1.10
	5	*F*1	0.22	0.30	0.42	0.51	0.58	0.62	0.65	0.66	0.67	0.68
		δ*F*1	0.00	36.80	85.48	46.62	9.63	2.94	1.18	0.35	0.39	0.40
senseit_sei	2	*F*1	0.60	0.60	0.61	0.61	0.61	0.61	0.61	0.61	0.61	0.62
		δ*F*1	171.08	171.28	173.05	174.66	173.47	165.56	87.27	49.27	25.05	17.71
	5	*F*1	0.62	0.64	0.64	0.64	0.64	0.65	0.64	0.64	0.65	0.66
		δ*F*1	180.46	185.59	186.24	181.20	66.10	28.37	11.36	3.14	1.37	0.60

**Figure 16:**
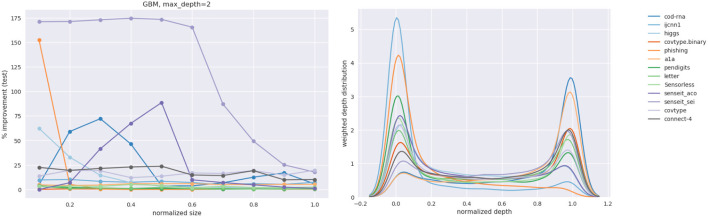
GBM with *max*_*depth* = 2. Size is the number of rounds.

**Figure 17:**
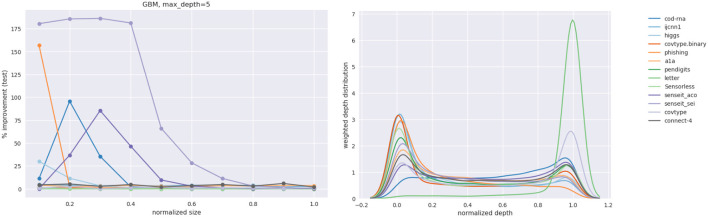
GBM with *max*_*depth* = 5. Size is the number of rounds.

*See Table A1 for complete data, Figures 16, 17 for plots*.

*Both F1_new_ and δF1 are shown. Please see text for explanation of the highlighting scheme*.

Figures 16, 17 show the improvement and depth distribution plots for the GBMs with *max*_*depth* = 2 and *max*_*depth* = 5, respectively.

The cells highlighted in blue in Table 4 are where the GBM with *max*_*depth* = 2 showed a larger improvement than a GBM with *max*_*depth* = 5 for the same number of boosting rounds. The cells highlighted in red exhibit the opposite case. Clearly, both factors manifest themselves. Comparing the relative improvement plots in Figures 16, 17, we see that improvements continue up to larger sizes when *max*_*depth* = 2 (also evident from Table 4). This is not surprising: we expect a stronger model to extract patterns from data at relatively smaller sizes, compared to a weaker model.

Observe that in Table 4, for the same number of boosting rounds, the new scores *F*1_*new*_ for the weaker GBMs are up to as large (within some margin of error) as the scores for the stronger GBMs. This is to be expected since our sampling technique diminishes the gap between representational and effective capacities (when such a gap exists); it does not improve the representational capacity itself. Hence a weak classifier using our method is not expected to outperform a strong classifier that is also using our method.

The depth distribution plots for the GBMs show a familiar pattern: high concentration at the root or the leaves. Also, similar to DTs and LPMs, the greatest improvements for a dataset mostly occur at relatively smaller model sizes (see Table A1).

#### 4.2.4. Summary

Summarizing our analysis above:

1. We see significant improvements in the *F*1 score across different combinations of model families, model sizes and datasets with different dimensionalities and label distributions.
2. Since in the DT experiments, we have multiple datasets for which we reached the optimal tree size, we were able to empirically validate the following related key hypotheses:
  - With larger model sizes the optimal distribution tends toward the original distribution. This is conveniently indicated with *p*_*o*_ → 1 as η increases.
  - There is model size η′, beyond which δ*F*1 ≈ 0%.
3. For all the model families experimented with—DTs, LPMs, GBMs (results in Table A1)—the greatest improvements are seen for relatively smaller model sizes.
4. In the case of DTs, the improvements are, in general, higher with multiclass than binary datasets. We do not see this disparity for LPMs. We believe this happens because of our subjective notion of size: in the case of DTs there is a single tree to which the size constraint applies, making the baseline easier to beat for multiclass problems; while for LPMs it applies to each *one-vs.-rest* linear model.
  Its harder to characterize the behavior of the GBMs in this regard, since while the base classifiers are DTs, each of which is a multiclass classifier, a GBM maybe comprised of multiple DTs.
5. The GBM experiments give us the opportunity to study the effect of using model families, F, of different strengths. We make the following observations:
  - We see both these factors at work: (1) a weaker model family has an easier baseline to beat, which may lead to higher δ*F*1 scores relative to using a stronger model family (2) a stronger model family is likely to make better use of the optimal distribution, which may lead to higher δ*F*1 scores relative to using a weaker model family.
  - For a stronger model family, the benefit of using our algorithm diminishes quickly as model size grows.
  - While the improvement δ*F*1 for a weaker family may exceed one for a stronger family, the improved score *F*1_*new*_ may, at best, match it.
6. The depth distribution seems to favor either nodes near the root or the leaves, and this pattern is consistent across learning algorithms and datasets.

Given our observations, *we would recommend using our approach as a pre-processing step for any size-limited learning*, regardless of whether the size is appropriately small for our technique to be useful or not. If the size is large, then our method will return to the original sample anyways.

## 5. Discussion

In addition to empirically validating our algorithm, the previous section also provided us with an idea of the kind of results we might expect of it. Using that as a foundation, we revisit our algorithm in this section, to consider some of our design choices and possible extensions.

### 5.1. Algorithm Design Choices

Conceptually, Algorithm 5 consists of quite a few building blocks. Although we have justified our implementation choices for them in section 3, it is instructive to look at some reasonable alternatives.

1. Since we use our depth distribution to identify the value of a depth ∈ ℤ_≥0_, a valid question is why not use a discrete distribution, e.g., a multinomial? Our reason for using a continuous distribution is that we can use a fixed number of optimization variables to characterize a density tree of *any* depth, with just an additional step of discretization. Also, recall that the depth distribution applies to *all* density trees in the forest *B*, each of which may have a different depth. A continuous distribution affords us the convenience of not having to deal with them individually.
2. A good candidate for the depth distribution is the *Pitman-Yor* process (Pitman and Yor, 1997)—a two-parameter generalization of the DP (recall, this has one parameter: α). Considering our results in Figures 14–17, where most depth distributions seem to have up to two dominant modes, we did not see a strong reason to use a more flexible distribution at the cost of introducing an optimization variable.
3. We considered using the *Kumaraswamy* distribution (Kumaraswamy, 1980) instead of *Beta* for the mixture components. The advantage of the former is its *cumulative distribution function* maybe be expressed as a simple formula, which leads to fast sampling. However, our tests with a Python implementation of the function showed us no significant benefit over the *Beta* in the *SciPy* package, for our use case: the depth distribution is in one dimension, and we draw samples in batches (all samples for a component are drawn simultaneously). Consequently, we decided to stick to the more conventional *Beta* distribution^8^.

### 5.2. Extensions and Applications

Our algorithm is reasonably abstracted from low level details, which enables various extensions and applications. We list some of these below:

1. **Smoothing**: We had hinted at alternatives to Laplace smoothing in section 3.3. We discuss one possibility here. Assuming our density tree has *n* nodes, we let *S* ∈ ℝ^*n*×*n*^ denote a pairwise *similarity matrix* for these nodes, i.e., [*S*]_*ij*_ is the similarity score between nodes *i* and *j*. Let *P* ∈ ℝ^1×*n*^ denote the base (i.e., before smoothing) probability masses for the nodes. Normalizing $P\times S^{k},k\in {\mathbb{Z}}_{\geq 0}$ gives us a smoothed *pmf* that is determined by our view of similarity between nodes. Analogous to *transition matrices*, the exponent *k* determines how diffuse the similarity is; this can replace λ as an optimization variable.
  The ability to incorporate a node similarity matrix opens up a wide range of possibilities, e.g., *S* might be based on the *Wu-Palmer* distance (Wu and Palmer, 1994), *SimRank* (Jeh and Widom, 2002), or *Random Walk with Restart (RWR)* (Pan et al., 2004).
2. **Categorical variables**: We have not explicitly discussed the case of categorical features. There are a couple of ways to handle data with such features:
  - The density tree may directly deal with categorical variables. When sampling uniformly from a node that is defined by conditions on both continuous and categorical variables, we need to combine the outputs of a continuous uniform sampler (which we use now) and a discrete uniform sampler (i.e., multinomial with equal masses) for the respective feature types.
  - We could create a version of the data with one-hot encoded categorical features for constructing the density tree. For input to $train_{F}\left(\;\right)$ at each iteration, we transform back the sampled data by identifying values for the categorical features to be the maximums in their corresponding sub-vectors. Since the optimizer already assumes a black-box $train_{F}\left(\;\right)$ function, this transformation would be modeled as a part of it.
3. **Model compression**: An interesting possible use-case is model compression. Consider the column *boostinground* = 1 for the *senseit_sei* dataset in Table 4. Assuming the base classifiers have grown to their *max*_*depths*, the memory footprint in terms of nodes for the GBMs with *max*_*depth* = 2 and *max*_*depth* = 5 are 2^2^ + 1 = 5 and 2^5^ + 1 = 33, respectively.
  Replacing the second model (larger) with the first (small) in a memory constrained system reduces footprint by (33−5)/33 = 85% at the cost of changing the *F*1 score by (0.60−0.62)/0.62 = −3.2% only.
  Such a proposition becomes particularly attractive if we look at the baseline scores, i.e., accuracies on the original distribution. For the larger model, *F*1_*baseline*_ = *F*1_*new*_/(1 + δ*F*1/100) = 0.62/(1 + 1.8046) = 0.22. If we replace this model with the smaller model enhanced by our algorithm, we not only reduce the footprint but actually *improve* the *F*1 score by (0.60−0.22)/0.22 = 173.7%!
  We precisely state this application thus: our algorithm may be used to identify a model size η_*e*_ (subscript “e” for “equivalent”) in relation to a size η > η_*e*_ such that:
  (16) $$\begin{array}{r} accuracy\left(train_{F}\left(p_{\eta_{e}}^{*},\eta_{e}\right),p\right)\approx accuracy\left(train_{F}\left(p,\eta \right),p\right) \end{array}$$
4. **Segment analysis**: Our sampling operates within the bounding box *U* ⊂ ℝ^*d*^; in previous sections, *U* was defined by the entire input data. However, this is not necessary: we may use our algorithm on a subset of the data *V* ⊂ *U*, as long as *V* is a hyperrectangle in ℝ^*d*^′, *d*′ ≤ *d*. This makes our algorithm useful for applications like *cohort analysis*, common in marketing studies, where the objective is to study the behavior of a segment—say, based on age and income—within a larger population. Our algorithm is especially appropriate since traditionally such analyses have emphasized interpretability.
5. **Multidimensional size**: The notion of size need not be a scalar. Our GBM experiments touch upon this possibility. The definition of size only influences how the call to $train_{F}\left(\;\right)$ *internally executes*; Algorithm 5 itself is agnostic to this detail. This makes our technique fairly flexible. For example, it is easy in our setup to vary both *max*_*depth* and *number of boosting rounds* for GBMs.
6. **Different optimizers**: As mentioned in section 3.1.2, the fact that our search space has no special structure implies the workings of the optimizer is decoupled from the larger sampling framework. This makes it easy to experiment with different optimizers. For example, an interesting exercise might be to study the effect of the hybrid optimizer *Bayesian Optimization with Hyperband (BOHB)* (Falkner et al., 2018) when $train_{F}\left(\;\right)$ is an iterative learner; BOHB uses an early stopping strategy in tandem with Bayesian Optimization.
7. **Over/Under-sampling**: As the range of the sample size parameter *N*_*s*_ is set by the user, the possibility of over/under-sampling is subsumed by our algorithm. For instance, if our dataset has 500 points, and we believe that sampling up to 4 times might help, we can simply set *N*_*s*_ ∈ [500,2,000]. Over/Under-sampling need not be explored as a separate strategy.

## 6. Conclusion

Our work addresses the trade-off between interpretability and accuracy. The approach we take is to identify an optimal training distribution that often dramatically improves model accuracy for an arbitrary model family, especially when the model size is small. We believe this is the first such technique proposed. We have framed the problem of identifying this distribution as an optimization problem, and have provided a technique that is empirically shown to be useful across multiple learning algorithms and datasets. In addition to its practical utility, we believe this work is valuable in that it challenges the conventional wisdom that the optimal training distribution is the test distribution.

A unique property of our technique is that beyond a pre-processing step of constructing a DT, which we refer to as a density tree, the number of variables in the core optimization step does not depend on the dimensionality of the data; it uses a fixed set of eight variables. The density tree is used to determine a feasible space of distributions to search through, making the optimization efficient. Our choice of using DTs is innovative since while all classifiers implicitly identify boundaries, only few classifiers like DTs, rules, etc., can explicitly indicate their locations in the feature space. We have also discussed how our algorithm may be extended in some useful ways.

We hope that the results presented here would motivate a larger discussion around the effect of training distributions on model accuracy.

## Data Availability Statement

The datasets analyzed for this study can be found on the LIBSVM website at https://www.csie.ntu.edu.tw/~cjlin/libsvmtools/datasets/multiclass.html and https://www.csie.ntu.edu.tw/~cjlin/libsvmtools/datasets/binary.html.

## Author Contributions

AG and BR have jointly formulated the problem, worked on certain aspects of the representation and designed experiments. AG has additionally worked on practical aspects of the representation, and executed the experiments.

### Conflict of Interest

The authors declare that the research was conducted in the absence of any commercial or financial relationships that could be construed as a potential conflict of interest.

## Footnotes

^1^https://blogs.wsj.com/cio/2018/05/11/bank-of-america-confronts-ais-black-box-with-fraud-detection-effort/

^2^https://www.darpa.mil/program/explainable-artificial-intelligence

^3^https://www.propublica.org/article/machine-bias-risk-assessments-in-criminal-sentencing, https://www.propublica.org/article/how-we-analyzed-the-compas-recidivism-algorithm

^4^The optimizer we use, TPE, *can* handle conditional spaces. However, as mentioned, our goal is flexibility in implementation.

^5^We justify this name by noting that there is more than one multivariate generalization of the *Beta*: the *Dirichlet distribution* is a popular one, but there are others (e.g., Olkin and Trikalinos, 2014).

^6^We use this term since this helps us define a *pdf* over the input space ℝ^*d*^. We don't abbreviate this term to avoid confusion with “DT.” DT always refers to a decision tree in this work, and the term “density tree” is used as-is.

^7^We later observe from our experiments that this upper bound is sufficient since nearly all depth distributions have at most 2 dominant components (see Figures 14–17).

^8^Interestingly, another recent paper on interpretability does use the Kumaraswamy distribution (Bastings et al., 2019).
