# Supplementary material for: Interpretability With Accurate Small Models
Source: Front Artif Intell. 2020 Feb 25;3:3. doi: 10.3389/frai.2020.00003 (PMC7861231; doi:10.3389/frai.2020.00003)
Supplement: Supplementary file 1 [file Data_Sheet_1.pdf]

A Appendix

A.1 Implementation Details

Setting the lower bound of the N_s parameter (see Section 3.3 in the main paper) to ensure statistical significance is not sufficient in itself. Since our sample comes from both the density trees and the original training data, we must ensure these samples lead to statistically significant results *individually*.

In order to do so the our implementation internally adjusts the quantity p_o. Recall that $p_o \in [0, 1]$. A low value of p_o can result in a small sample of size $p_o N_s$ from the original training data, while a high value of p_o might result in a small sample of size $(1 - p_o)N_s$ from the density trees. Interestingly however, $p_o = 0$ and $p_o = 1$ should be allowed as valid values, since the sample is then drawn from only one of the sources and is therefore not small!

The adjustment we make is shown in Figure 1. The x-axis shows the current value of p_o, the y-axis shows what the sampler sees.

Below a user specified threshold for p_o, it is adjusted to $p_o = 0$. Beyond a certain user specified threshold, it is adjusted to $p_o = 1$. The lack of smoothness or differentiability of the adjustment does not impact our optimization, since a BO would construct its version of the objective function anyway.

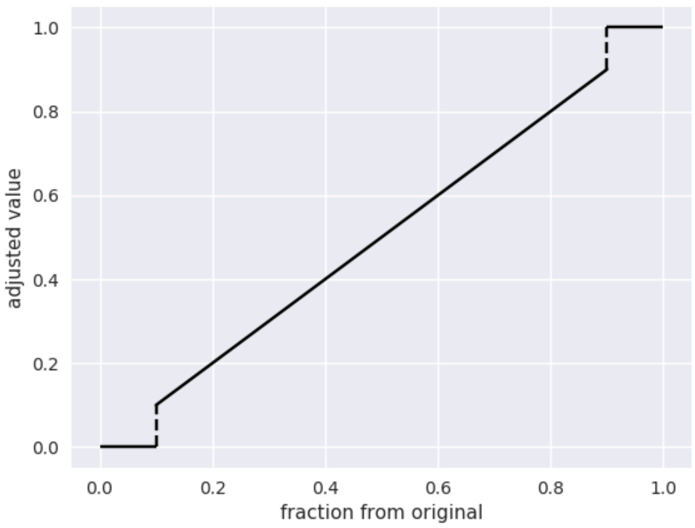

Figure 1: Adjustments to p_o.

Table A1: $F1_{new}$ and $\delta F1$ scores are shown for GBM models with $max\_depth = 2$ and $max\_depth = 5$. All scores are averaged over five runs. Note that the notion of statistical significance only applies to the $\delta F1$ entries, since we use a *paired t-test*; for these rows, bold font denotes statistical significance, and normal font denotes the entry is not statistically significant. Underlined entries denote the best improvement for a dataset.

datasets	max depth	boosting rounds = / score type	1	2	3	4	5	6	7	8	9	10
cod-rna	2	$F1$	0.42	0.64	0.69	0.70	0.70	0.71	0.74	0.78	0.82	0.84
		$\delta F1$	4.78	**59.01**	**72.16**	**46.26**	**3.65**	**3.67**	**6.69**	**12.45**	**16.93**	**5.60**
	5	$F1$	0.44	0.78	0.83	0.84	0.86	0.87	0.88	0.88	0.89	0.89
		$\delta F1$	**11.24**	**95.76**	**35.47**	**2.13**	**0.63**	**0.46**	**0.61**	0.20	**0.19**	0.33
ijcnn1	2	$F1$	0.71	0.71	0.71	0.71	0.72	0.72	0.71	0.71	0.71	0.72
		$\delta F1$	**9.61**	**10.12**	**8.21**	**7.33**	**8.34**	**7.01**	**4.70**	**5.91**	**5.22**	**7.22**
	5	$F1$	0.77	0.77	0.77	0.78	0.78	0.79	0.79	0.79	0.79	0.80
		$\delta F1$	**4.88**	**3.94**	**3.57**	**3.47**	**3.32**	**3.99**	**3.85**	**3.88**	**2.03**	**3.04**
higgs	2	$F1$	0.61	0.62	0.63	0.63	0.63	0.63	0.63	0.64	0.64	0.64
		$\delta F1$	**62.31**	**32.90**	**14.43**	**6.90**	**3.12**	**2.29**	**2.26**	**1.32**	**1.79**	**1.90**
	5	$F1$	0.62	0.64	0.64	0.65	0.66	0.66	0.67	0.67	0.67	0.68
		$\delta F1$	**29.91**	**11.54**	**3.35**	**0.97**	**1.34**	**1.14**	0.06	**1.08**	**0.78**	**0.48**
covtype.binary	2	$F1$	0.73	0.73	0.73	0.73	0.74	0.74	0.74	0.74	0.74	0.74
		$\delta F1$	0.14	**0.55**	**0.51**	**0.68**	**0.75**	**0.91**	**0.65**	**0.69**	**0.46**	**0.74**
	5	$F1$	0.76	0.76	0.77	0.76	0.76	0.77	0.77	0.77	0.77	0.77
		$\delta F1$	**1.15**	**0.76**	0.78	0.49	**0.99**	**0.76**	**0.50**	0.07	0.15	0.06
phishing	2	$F1$	0.90	0.91	0.91	0.91	0.91	0.91	0.91	0.91	0.91	0.91
		$\delta F1$	**152.30**	**5.18**	0.29	0.02	0.01	0.03	**0.36**	**0.40**	**0.32**	**0.21**
	5	$F1$	0.92	0.93	0.93	0.93	0.93	0.93	0.93	0.93	0.93	0.94
		$\delta F1$	**156.68**	**1.88**	**1.08**	**1.26**	**0.89**	**0.72**	**0.57**	**0.56**	**0.67**	**0.43**
a1a	2	$F1$	0.71	0.72	0.72	0.72	0.73	0.73	0.73	0.73	0.73	0.74
		$\delta F1$	**4.69**	**3.99**	**4.51**	**5.87**	**5.89**	**5.91**	**4.97**	**4.92**	**5.12**	**5.09**
	5	$F1$	0.74	0.74	0.75	0.75	0.74	0.74	0.75	0.75	0.75	0.76
		$\delta F1$	**3.91**	**2.59**	**2.91**	**3.74**	**2.79**	**2.75**	**3.43**	**3.07**	**3.65**	**3.59**
pendigits	2	$F1$	0.76	0.80	0.81	0.82	0.82	0.83	0.83	0.84	0.84	0.84
		$\delta F1$	**3.76**	**1.96**	**1.47**	**0.70**	**0.91**	**0.64**	**0.40**	**0.47**	**0.47**	**0.67**
	5	$F1$	0.92	0.94	0.94	0.95	0.95	0.95	0.95	0.96	0.96	0.96
		$\delta F1$	0.17	0.21	0.18	0.17	0.05	0.02	**0.11**	0.00	0.02	0.07
letter	2	$F1$	0.53	0.58	0.59	0.61	0.61	0.62	0.63	0.63	0.63	0.64
		$\delta F1$	**3.10**	**0.99**	**1.47**	**0.99**	**2.10**	0.70	**1.17**	**0.40**	**0.79**	**0.43**
	5	$F1$	0.71	0.76	0.77	0.78	0.79	0.80	0.80	0.80	0.81	0.82
		$\delta F1$	**1.05**	0.11	0.00	0.03	0.00	0.00	0.00	0.10	0.00	0.00
Sensorless	2	$F1$	0.76	0.77	0.78	0.80	0.80	0.80	0.81	0.81	0.81	0.81
		$\delta F1$	**3.19**	**3.36**	**3.11**	**5.05**	**4.13**	**1.75**	**3.22**	**1.98**	**1.90**	**2.43**
	5	$F1$	0.91	0.92	0.93	0.94	0.94	0.94	0.94	0.95	0.95	0.95
		$\delta F1$	0.29	0.26	**0.16**	**0.41**	0.00	0.18	0.37	**0.30**	0.00	**0.26**
senseit_aco	2	$F1$	0.22	0.24	0.31	0.37	0.52	0.59	0.61	0.62	0.63	0.63
		$\delta F1$	0.00	6.81	**41.41**	**67.44**	**88.57**	**9.76**	**6.97**	**4.73**	**2.34**	**1.10**
	5	$F1$	0.22	0.30	0.42	0.51	0.58	0.62	0.65	0.66	0.67	0.68
		$\delta F1$	0.00	**36.80**	**85.48**	**46.62**	**9.63**	**2.94**	**1.18**	0.35	0.39	**0.40**
senseit_sei	2	$F1$	0.60	0.60	0.61	0.61	0.61	0.61	0.61	0.61	0.61	0.62
		$\delta F1$	**171.08**	**171.28**	**173.05**	**174.66**	**173.47**	**165.56**	**87.27**	**49.27**	**25.05**	**17.71**
	5	$F1$	0.62	0.64	0.64	0.64	0.64	0.65	0.64	0.64	0.65	0.66
		$\delta F1$	**180.46**	**185.59**	**186.24**	**181.20**	**66.10**	**28.37**	**11.36**	**3.14**	**1.37**	**0.60**
covtype	2	$F1$	0.41	0.42	0.41	0.41	0.41	0.40	0.41	0.40	0.40	0.40
		$\delta F1$	**13.72**	**19.49**	**19.02**	**12.06**	**13.47**	**16.84**	**16.32**	**19.27**	**14.65**	**19.58**
	5	$F1$	0.47	0.48	0.48	0.48	0.49	0.49	0.50	0.50	0.49	0.50
		$\delta F1$	0.68	2.69	0.16	1.19	**1.51**	0.54	1.17	0.72	0.44	1.39
connect-4	2	$F1$	0.43	0.44	0.45	0.45	0.47	0.46	0.47	0.47	0.47	0.47
		$\delta F1$	**22.44**	**19.47**	**21.39**	**22.89**	**23.65**	**14.75**	**14.16**	**19.16**	**9.82**	**10.07**
	5	$F1$	0.48	0.49	0.50	0.51	0.51	0.52	0.52	0.53	0.53	0.53
		$\delta F1$	**4.30**	**5.36**	**2.70**	**4.75**	**1.93**	**3.51**	**4.74**	**2.98**	**5.91**	**1.95**

A.2 GBM Results

Table A1 represents the improvements seen using GBMs where we have $max\_depth = 2$ or $max\_depth = 5$ for the base classifier trees. This is an expanded version of data presented in Table 4 in the main paper. Note here that much like DTs and LPMs, we see the largest $\delta F1$ values typically for relatively smaller model sizes.

A.3 Harmonic Numbers

The Nth harmonic number is defined as:

$$H_N = 1 + \frac{1}{2} + \frac{1}{3} + ... + \frac{1}{N} = \sum_{k=1}^{N} \frac{1}{k} \quad (1)$$

Clearly $H_N \propto N$, since increasing N adds positive terms to H_N. Figure 2 shows the relationship of H_N and N for $N = 1, 2, ..., 100$

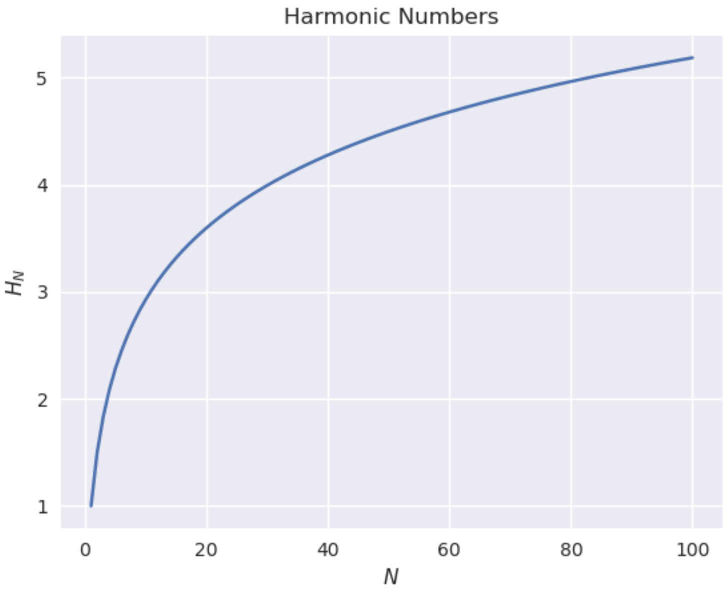

Figure 2: Variation of H_N with increasing N.
